# Future Range Expansions of Invasive Wasps Suggest Their Increasing Impacts on Global Apiculture

**DOI:** 10.3390/insects15070546

**Published:** 2024-07-19

**Authors:** Xueyou Zhang, Peixiao Nie, Xiaokang Hu, Jianmeng Feng

**Affiliations:** 1 College of Agriculture and Biological Science, Dali University, Dali 671003, China; zhangxueyou0604@163.com (X.Z.); niepeixiao929@163.com (P.N.); 2 Cangshan Forest Ecosystem Observation and Research Station of Yunnan Province, Dali University, Dali 671003, China

**Keywords:** future climate changes, global apiculture, impacts, invasive wasps, range expansions

## Abstract

**Simple Summary:**

Invasive wasps can have huge impacts on global apiculture. Here, we developed 12 range dynamic models to globally calibrate the future range dynamics and invasion hotspots of 12 major invasive Vespidae wasp species through a unified approach and evaluated their future impacts on apiculture worldwide. We detected increases in their habitat suitability in most parts of the globe and substantial range expansions, both mainly driven by future climatic changes. We also identified those invasive wasp species projected to have the largest potential ranges, highest range expansion ratios, and largest centroid shifts, as well as the invasion hotspots of all major invasive wasp species in the future. In summary, the increasing habitat suitability and range dynamics of invasive wasps indicate that global apiculture will likely face greater adverse impacts in the future. Therefore, our study provides important and novel information for combatting invasions of wasps and mitigating their expected impacts on global apiculture.

**Abstract:**

Until now, no study has examined the future range dynamics of major invasive wasp species to assess their future impacts on global apiculture. Here, we developed 12 species distribution models to calibrate the future range dynamics of 12 major invasive Vespidae wasp species under a unified framework. An increase in their habitat suitability was identified in more than 75% of global land. Substantial range expansions were detected for all 12 species, and they were primarily induced by future climate changes. Notably, *Polistes dominula* and *Vespa crabro* had the largest potential ranges under all scenarios, suggesting their greater impact on global apiculture. *Polistes chinensis* and *Vespa velutina nigrithorax* had the highest range expansion ratios, so they warrant more urgent attention than the other species. *Polistes versicolor* and *P. chinensis* are expected to exhibit the largest centroid shifts, suggesting that substantial shifts in prioritizing regions against their invasions should be made. Europe and the eastern part of the USA were future invasion hotspots for all major invasive wasp species, suggesting that apiculture might face more pronounced threats in these regions than in others. In conclusion, given their substantial range shifts, invasive wasps will likely have increasingly negative impacts on global apiculture in the future.

## 1. Introduction

Beekeeping is important for securing food, poverty reduction, health, environmental protection, and plant pollination [1]. However, numerous biotic and abiotic factors have emerged in recent years to challenge this important practice [2]. In particular, the impacts of Vespidae wasps on apiculture have garnered much attention, probably because they prey on bees (Apidae), leading to destructive impacts on global apiculture [3,4,5,6]. With their larger bodies compared to bees, thick chitinous armors to fend off attacks, powerful mandibles, and deadly stings, invasive wasps pose a serious threat to various species of honey bees [7,8,9]. Additionally, although their establishment time varies with species, they can establish viable populations in very short periods. For example, *Vespa velutina* established its population ca. ten years after its introduction into France [10], and *Polistes dominula* established its population in North America in less than twenty years [11,12]. Therefore, numerous studies have shown that most invasive wasps could strongly decrease honey bee colonies, resulting in huge economic losses for global apiculture [5,13,14,15,16,17,18]. Generally, they kill bees and steal honey from beehives, resulting in a direct financial loss to global apiculture, and they can also cause delays in the extraction of honey, requiring beekeepers to spend time and money on control procedures. Additionally, they can compete with bees for some food sources, causing food shortages and population reduction in bee colonies [9,13,19]. For example, the main portion of dietary protein for *Vespa velutina nigrithorax*, an invasive wasp, comes from Western honey bees (*Apis mellifera*) in Europe [3], and its impacts include reducing hive sizes by 50% [16,17], damage to 18–30% of bee hives [5], and reductions in bee-derived products [16,17,18]. In France alone, the corresponding economic losses range from EUR 100 million per year to EUR 1.5 billion per year [20]. All of these observations indicate that strict and highly efficient management and prevention strategies are imperative to control wasp invasions [6,21]. However, we have to acknowledge that the impacts of invasive wasps vary with species. For example, compared with *Polistes* wasps, Vespinae species have shown stronger impacts on beekeeping [22,23]. Also, invasive wasps can compete with local wasp species already existing in a locality. For example, invasive *Vespa velutina* in Europe could significantly reduce the abundance of *V. crabro* through food resource and habitat competition when the former has a high population density [24]. 

It is reasonable to assume that detecting priority regions or potential ranges of invasive wasps should be a key component of strategies devised to counter their spread. Accordingly, in recent decades, we have paid much attention to the range dynamics of invasive wasps [25,26,27,28,29]. For example, Howse et al. detected substantial range expansion by *Polistes dominula*, one of the major invasive wasp species, and uncovered its invasion hotspots in eastern Argentina, central Chile, southern Brazil, and parts of Uruguay [30]. More recently, Nie et al. projected the future range expansion of invasive *Vespa velutina* in Europe, detecting priority regions in Iceland, the British Isles, Switzerland, Liechtenstein, Austria, Poland, Germany, and Northern Europe [31]. These studies have certainly enhanced our understanding of the range shifts of invasive wasps under future scenarios. That is, the future-climate-change-induced range expansion of invasive wasps could pose an increasing threat to apiculture in the studied areas, though we have to acknowledge that range expansion may result in asynchrony in predator–prey interactions [32], probably resulting in a decrease in apiaries’ economic losses in their indigenous localities. Here, we note that most of the work that has been carried out has lacked a unified approach to investigating the range shifts of invasive wasps with respect to candidate factors, spatial scales, and candidate algorithms. This makes it difficult to fairly compare studies. For example, Masciocchi and Corley used climatic and biological data as predictors to project the potential ranges of *Vespula germanica* in Argentina via CLIMEX models [22], while Verdasca et al. instead used climatic and anthropogenic variables to predict the potential ranges of *Vespa velutina* in Portugal by fitting generalized linear mixed models [33]. 

Numerous studies have projected the range shifts of invasive wasps and identified their invasion hotspots or priority regions [29,34,35,36,37]. However, we noted that few of the relevant studies investigated overlapping invasion hotspots of the major invasive wasp species at a global scale [25,30,38,39]. This implies a need to detect priority regions, i.e., those regions in which the threats from invasive wasps are greater. Furthermore, although invasive wasps can have substantial impacts on global apiculture [4,5,6], few studies have globally explored the future range dynamics of all the major invasive wasp species and thereby assessed their future impacts on global apiculture. 

Climatic changes are some of the factors most closely associated with the range dynamics of invasive wasps. Lioy et al. detected a strong influence of climatic variables on the ranges of *Vespa orientalis* in Europe [30]. Earlier, Moo-Llanes projected future-climate-change-induced range shifts of the invasive Asian giant hornet (*Vespa mandarinia*) into central and northern USA and other parts of the Americas [40]. But, to the best of our knowledge, few studies have used a unified approach (such as unified candidate predictors and algorithms) to investigate the role of anticipated climatic changes in the range shifts of all major invasive wasp species at a global scale.

Besides changes in climate, land-use factors are also expected to be closely associated with the range dynamics of invasive wasps. For instance, Verdasca et al. detected close associations between anthropogenic factors and the dispersal of invasive wasps and found that these factors played strong roles in their range dynamics [33]. Another study found that the increasing extent of agricultural land played a pivotal role in the range expansion of *Vespa velutina nigrithorax* into the Iberian Peninsula [25]. Several studies have argued that, compared to anthropogenic factors, climatic changes play a stronger role in the range dynamics of invasive wasps [25,31,33]. However, a study by Sumner et al. in the UK showed that, relative to climatic factors, land-use variables exerted stronger influences on the range shifts of three invasive wasp species, i.e., *Vespula vulgaris*, *V. germanica*, and *Vespa crabro* [26]. Therefore, the relative roles of climatic and land-use factors in the range shifts of invasive wasps are in need of further investigation. 

Here, we aimed to use a unified approach to project the global range shifts of all major Vespidae invasive wasp species and thus calibrated their overlapped invasion hotspots, and we also assessed their impacts on global apiculture. We hypothesized that the relative role of climate vis-à-vis land-use factors in wasp range shifts might be species-specific. Our findings offer new and crucial information for devising efficient strategies against looming global invasions of invasive wasps and their future impacts on global apiculture.

## 2. Materials and Methods

### 2.1. Presence Records of the 12 Major Invasive Wasp Species

**Figure 1:**
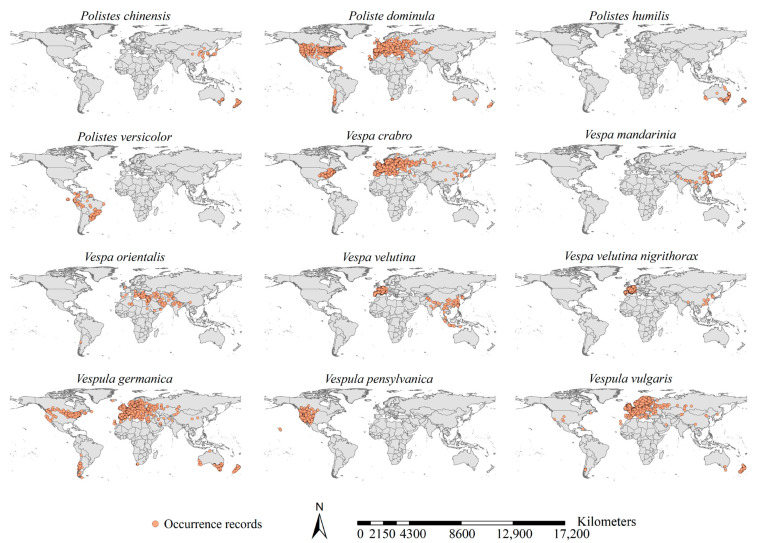
Occurrences of the 12 major invasive wasp species. Occurrences were retrieved from the Global Biodiversity Information Facility (www.gbif.org, accessed on 7 September 2023). A total of 19,196 occurrences were retrieved after spatial thinning.

Through an extensive literature survey via the Web of Science and Google Scholar, we compiled a checklist of the 12 major invasive wasp species belonging to the family Vespidae (Supplementary Materials S1, Figure 1). The unified source of their occurrence records was the Global Biodiversity Information Facility (www.gbif.org, accessed on 7 September 2023), one of the world’s largest online datasets for species occurrences, housing > 2.6 billion records from ca. 90,000 datasets, 2100 research institutions, and 10,000 academic papers containing species presence records. In total, we retrieved 270,574 records for the 12 major invasive wasp species; for each, we compiled a preliminary occurrence dataset. As suggested by Nie and Feng [31], for each species, we removed any of its occurrences for which there was uncertainty about geographical coordinates > 5 km. We then spatially thinned the occurrences with a diameter of 5 km for each species separately. Finally, we created a formal occurrence record dataset for each species, resulting in 19,196 records overall (Figure 1). 

### 2.2. Predictors in the Study

We compiled three categories of predictors to calibrate maps of habitat suitability and potential ranges for each invasive wasp species (Supplementary Materials S2), i.e., climate (19), land-use (8), and topographical predictors (3), amounting to 30 predictors (Supplementary Materials S2). The climate predictors consisted of eight temperature and eleven precipitation factors (Supplementary Materials S2). To obtain climate predictors under current scenarios, we retrieved the 1990–2020 average values of monthly temperature and precipitation variables with a 2.5-arc-minute spatial resolution from the Climate Research Division (CRU; https://crudata.uea.ac.uk/, accessed on 19 September 2023). Next, the R package ‘Biovarcs’ developed by Fick and Hijmans was applied to calculate 19 current climatic variables, which fully corresponded to variables in Worldclim [41]. We then obtained 19 future climate variables for 2100 from Worldclim [41]. These 19 future climate variables were calibrated by two complementary and reliable global circulation models (GCMs), i.e., MPI-ESM-HR (M) and FIO-ESM-2 (F) [42]. We adopted two future scenarios, i.e., Shared Socio-Economic Pathway (SSP) 126 (SSP126) and 585 (SSP585) scenarios, which respectively indicated the most optimistic and pessimistic SSP scenarios. Overall, therefore, we had five sets of climatic variables that corresponded to five scenarios: the current scenario, the SSP126 scenario generated by the FIO-ESM-2 GCM (F126), the SSP585 scenario generated by the FIO-ESM-2 GCM (F585), the SSP126 scenario generated by the MPI-ESM-HR GCM (M126), and the SSP585 scenario generated by the MPI-ESM-HR GCM (M585). 

The land-use predictors comprised eight land-use factors with a 0.25-arc-degree spatial resolution, downloaded from the Land-Use Harmonization online dataset (LUH2; https://luh.umd.edu/, retrieved on 25 September 2023), which were resampled to obtain a 2.5-arc-minute spatial resolution. These predictors were urbanized land, cropland, rangeland, unforested secondary land, unforested primary land, forested primary land, forested secondary land, as well as managed pasture (Supplementary Materials S2). The land-use factors included three datasets: the current scenario, the SSP126 scenario in 2100, and the SSP585 scenario in 2100. The topographical factors from Worldclim [40] included elevation, slope, and aspect (Supplementary Materials S2), all of which were calculated on the basis of a global digital elevation model (DEM) at a 0.5-arc-minute spatial resolution and then resampled to 2.5 arc minutes.

### 2.3. Statistical Analysis

For each species, we conducted a Pearson correlation analysis to gain correlation coefficients between each pair of the predictors. Then, a coefficient threshold of |0.7| was applied to detect the collinearity among the 30 predictors [31,43,44]. We also applied a paired-samples *t*-test to compare the ranges of the 12 invasive wasp species under current scenarios with those in the future.

### 2.4. Predictor Selection

For each species, a set of preliminary species distribution models (SDMs) was built to determine the importance of each factor through the jackknife technique (Supplementary Materials S2). For each species, only the predictors showing higher importance values were retained if we detected strong collinearity between any two predictors (Supplementary Materials S3). In the end, we inputted the retained predictors into the final species distribution models developed to determine the habitat suitability and ranges of our 12 target species separately, as well as the importance of each predictor. 

### 2.5. Developing the Species Distribution Models (SDMs)

We developed a total of 12 SDMs to project habitat suitability and ranges for the 12 invasive wasp species. For each species, we predicted its habitat suitability and ranges through an ensemble platform for species distribution models, i.e., the R package ‘Biomod2’ [45]. We applied the following 10 algorithms: a Surface Range Envelope, a Generalized Linear Model, Random Forest, Multiple Adaptive Regression Splines, Xtreme Gradient Boosting, Maximum Entropy Modeling, Classification Tree Analysis, Flexible Discriminant Analysis, an Artificial Neural Network, and a Generalized Boosting Model [45]. However, as suggested by Nie and Feng [43], only the algorithms showing a true skill statistic > 0.6 or an area under the curve > 0.8 could be inputted into the ensembled species distribution models (Supplementary Materials S4). Also, for each species, we conducted five-repetition selection to retrieve pseudo-absences (PAs) [46]: 1000 PAs were randomly and globally selected if the number of occurrences of an invasive wasp species was < 1000 or the number of PAs equaled that of the occurrence records. We applied a five-repetition cross validation to assess the performance of the SDMs, i.e., seven-tenths of the occurrences were randomly selected to build SDMs, with the remaining three-tenths used to evaluate the SDMs’ performance [47,48]. 

### 2.6. Examining Habitat Suitability Dynamics

For each species, we used occurrence records and current predictors to build a baseline SDM to predict habitat suitability maps under current scenarios. Then, the occurrence records and future predictors were inputted into the baseline SDM to project habitat suitability maps under future scenarios for each species individually. The dynamics of each invasive wasp species were estimated by subtracting the future habitat suitability maps from the current ones. We also created and calculated overlap indices of habitat suitability (*OIHS*s) (i.e., $OIHS=\sum_{i=1}^{N=12}HSi$) by overlapping the habitat suitability maps of the 12 invasive wasp species under each scenario separately. Finally, we calibrated the dynamic maps of the 12 invasive wasp species by subtracting their future *OIHS* maps from current ones. 

### 2.7. Examining the Range Dynamics

For each species, we utilized the maximum sensitivity–specificity sum threshold (MSS threshold) [49] to the five habitat suitability maps under the five scenarios and calibrated the potential ranges of each invasive wasp species. We constructed range shift models to explore the range shifts of the 12 invasive wasp species separately. To do this, we generated maps of the overlap index of potential range (*OIPR*, i.e., $OIPR=\sum_{i=1}^{N=12}PRi$) for each scenario, where *PR* is the potential range value of each species. The *OIPR* maps under the five scenarios were then compared.

We also created and estimated range expansion ratios (*RER*s) and range similarity indices (*RSI*s) to measure the range shifts of each invasive wasp species. The *RER*s were used to determine the range size shifts under current and future scenarios:

$$RER=\frac{RF}{RC},$$

where *RF* and *RC* are the future and current potential range areas, respectively.

We also used the *RSI* to examine the species’ range position shifts:

$$RSI=\frac{2RS}{RC+RF},$$

where *RC*, *RF*, and *RS* are the current ranges, the future ranges of each invasive wasp, and the ranges shared by *FR* and *CR*, respectively. An *RSI* < 0.5 indicates that the *RC* and the *RF* occupy similar range positions. 

To further probe the invasion risk posed by each wasp species, we also projected the ranges that they would potentially occupy or invade under each future scenario (the expanding range) according to a range overlap. Using the method to estimate *OIPR*, we also generated maps for the overlap indices of the expanding ranges of the 12 invasive wasp species for each scenario. 

To sum up, regions showing high overlap indices of habitat suitability, high overlap indices of potential ranges, and high overlap indices of expanding ranges were used to detect the invasion hotspots of the 12 invasive wasp species. We also used the sizes of their potential ranges and expanding ranges and their range expansion ratios and range similarity indices to gauge their range dynamics and thereby assess their impacts on global apiculture. 

## 3. Results

### 3.1. Reliability of the SDMs

Due to our multi-algorithm approach, our baseline SDMs for the invasive wasps showed high performance. The areas under the curves (AUCs) of the 12 species-specific baseline SDMs ranged from 0.991 to 0.999, averaging 0.995 ± 0.000 (Supplementary Materials S5). The values of their true skill statistics (TSSs) varied from 0.910 to 0.986, being 0.948 ± 0.030 on average (Supplementary Materials S5). The highest AUCs were identified for Polistes chinensis (0.999), *P. humilis* (0.999), and *Vespa velutina nigrithorax* (0.999), while the lowest were projected for *P. dominula* (0.991), *Vespula germanica* (0.991), and *Vespa orientalis* (0.991) (Supplementary Materials S5). The highest TSS scores were detected for *P. humilis* (0.985) and *Vespa velutina nigrithorax* (0.986), while the lowest were projected for *P. dominula* (0.910), *Vespula germanica* (0.914), and *Vespa orientalis* (0.910) (Supplementary Materials S5).

### 3.2. Main Predictors in the Baseline SDMs

**Figure 2:**
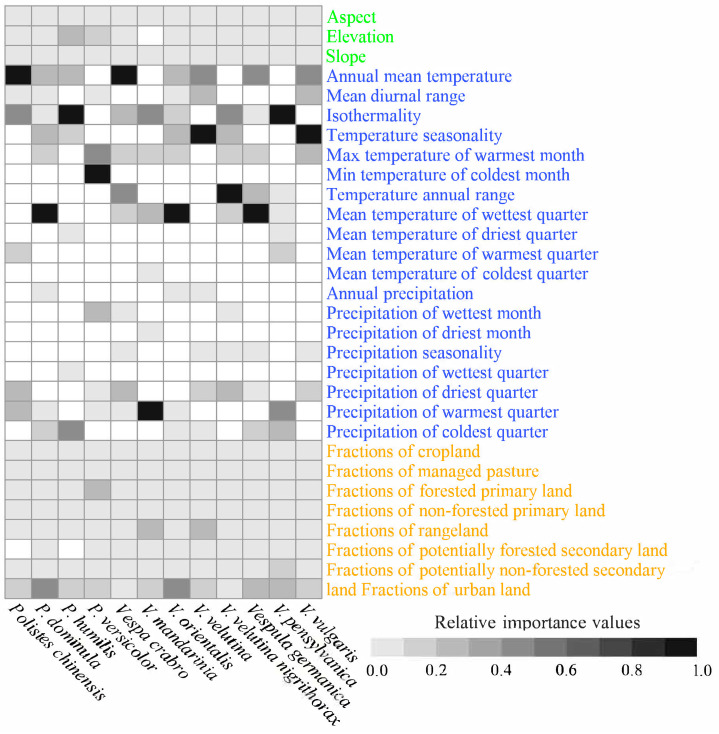
The importance of each predictor in the baseline models. Topographical, climatic, and land-use factors are shown in green, blue, and orange fonts, respectively. For each species, importance values were standardized according to the max–min method. The grayscale shading indicates the relative importance of each of the predictors, and the blanks indicate that the predictors were not inputted into the final models for the species.

The main predictors in the baseline SDMs were species-specific (Figure 2). For example, for *Polistes dominula*’s potential ranges, the predictors were the average temperature of the wettest season (with an importance equal to 0.071), the proportion of urban land (0.065), temperature seasonality (0.050), the mean annual temperature (0.031), and the maximum temperature of the warmest month (0.027) in that order. Those for *Polistes humilis* were isothermality (0.494), precipitation of the coldest season (0.092), annual mean temperature (0.027), elevation (0.024), and temperature seasonality (0.022) (Supplementary Materials S6, Figure 2). Climate predictors were distinguished by the highest importance values in all baseline SDMs for the 12 invasive wasp species (Supplementary Materials S6, Figure 2). Additionally, predictors in the categories of climate, land use, and topography appeared 50, 8, and 2 times, respectively, in the top-five-predictors lists for the 12 baseline SDMs (Supplementary Materials S6, Figure 2). In summary, compared with land-use and topographical predictors, climate predictors had much more important roles in shaping the range dynamics of the 12 invasive wasp species.

### 3.3. Habitat Suitability Patterns and Their Dynamics

The geographical patterns of habitat suitability were scenario-specific (Supplementary Materials S7). For example, under the M126 scenario, a high habitat suitability for *Polistes versicolor* was mainly projected in Nigeria, the Democratic Republic of Congo, Madagascar, Indonesia, Malaysia, and the southeastern portion of South America, whereas under the F585 scenario, high habitat suitability was mainly detected in scattered regions, including Colombia, southeastern Brazil, Tanzania, Madagascar, Indonesia, and Malaysia (Supplementary Materials S7). Geographical patterns of habitat suitability also varied with species identity. For instance, under the F126 scenario, a high habitat suitability for *Vespula vulgaris* was detected predominately in the European continent, while for *V. pensylvanica*, high habitat suitability indices were mainly limited to western USA (Supplementary Materials S7). 

**Figure 3:**
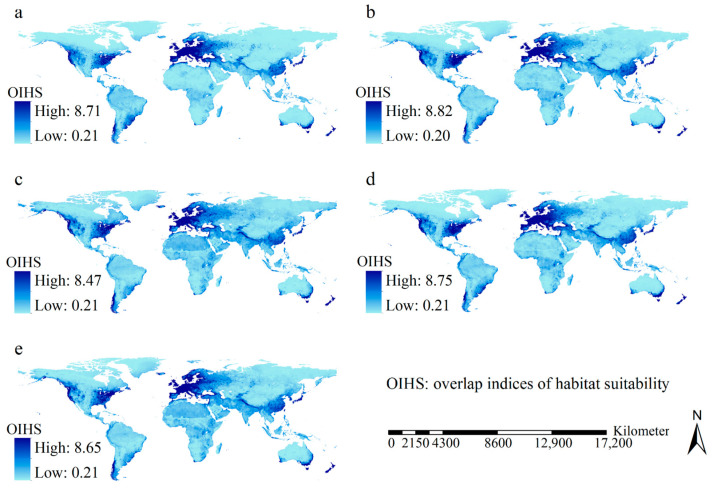
Overlap indices of habitat suitability of the 12 major invasive wasp species: (**a**) current scenario; (**b**) scenario of F126; (**c**) scenario of F585; (**d**) scenario of M126; (**e**) scenario of 585. High habitat suitability overlap indices in the five scenarios was detected in Europe, the eastern and western parts of the USA, and the southeastern part of Australia.

Although the maps for overlap indices of habitat suitability of the 12 invasive wasp species differed among the scenarios, they roughly displayed similar spatial patterns (Figure 3). The high overlap indices of habitat suitability occurred chiefly in Europe, eastern and western USA, and southeastern Australia. 

**Figure 4:**
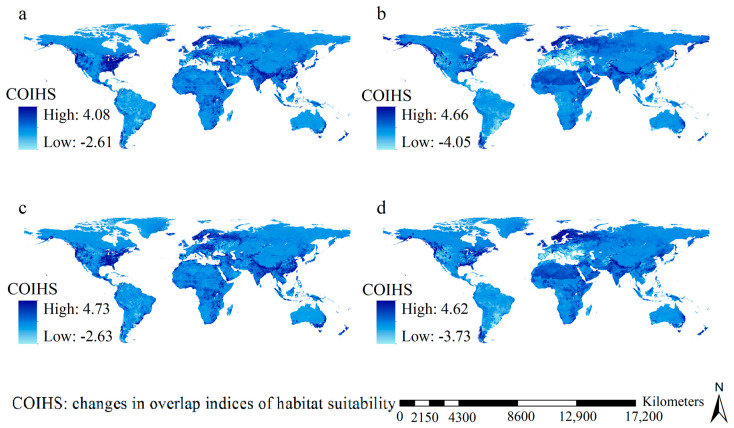
Changes in overlap indices of habitat suitability of 12 major invasive wasps. (**a**) F126; (**b**) F585; (**c**) M126; (**d**) M585. Considerable increases of overlap indices of habitat suitability under F126 and M126 were mainly detected in eastern coastline regions and the eastern part of USA, North Europe, East China, Nepal, New Zealand and southeastern coastlines of Australia. Large increases in the overlap indices of habitat suitability under the scenarios of M585 and F585 were primarily identified in the eastern part of North America, western coastline regions of the North America, North Europe, western part of Russia, Nepal, India, East China and the far-east regions of Russia.

Under the scenarios of M126 and F126, regions that featured considerable increases in overlap indices of habitat suitability were mainly identified in eastern coastline regions and the eastern part of the USA, in addition to Northern Europe, East China, Nepal, New Zealand, and southeastern coastline regions of Australia; in contrast, substantial decreases were detected in vast regions (Figure 4). Under the scenarios of M585 and F585, pronounced increases in overlap indices of habitat suitability were predicted in vast regions, including eastern North America, western coastline regions of North America, most of Northern Europe, and the western part of Russia, along with Nepal, India, East China, and the far-east regions of Russia, while marked decreases in overlap indices of habitat suitability were prevalent in Central and Southern Europe (Figure 4). Areas where the overlap indices of habitat suitability increased encompassed 106.93, 112.02, 104.36, and 106.89 million km^2^ under the F126 scenario, the M126 scenario, the F585 scenario, and the M585 scenario, respectively. In other words, increases in the overlap indices of habitat suitability in the future could be detected for 79.20%, 82.98%, 77.30%, and 79.18% of the global land area, respectively.

### 3.4. Invasive Wasp Species’ Potential Ranges

The MSS thresholds used to determine the potential ranges varied by scenario as well as species (Supplementary Materials S8). For instance, under the scenarios of F585 and M126, the MSS thresholds used to determine the potential ranges of *Polistes versicolor* were 0.31 and 0.69, respectively. For the ranges of *Vespula germanica* and *Vespula pensylvanica*, the MSS values under the F585 scenario were 0.69 and 0.29, respectively. Overall, the MSS thresholds varied from 0.27 to 0.84 (Supplementary Materials S8). The largest MSSs emerged for Vespula vulgaris (0.84 and 0.81, respectively, under the scenarios of M126 and F126), *Vespa mandarinia* (0.81 under the scenario of F126), and *Vespa velutina nigrithorax* (0.82 in the current period). Conversely, the smallest MSSs were mainly projected for *Polistes versicolor* (0.31 under the F585 scenario), *Vespula pensylvanica* (0.29 under F585; 0.31 under M585), and *Vespa velutina nigrithorax* (0.27 under F585) (Supplementary Materials S8).

**Figure 5:**
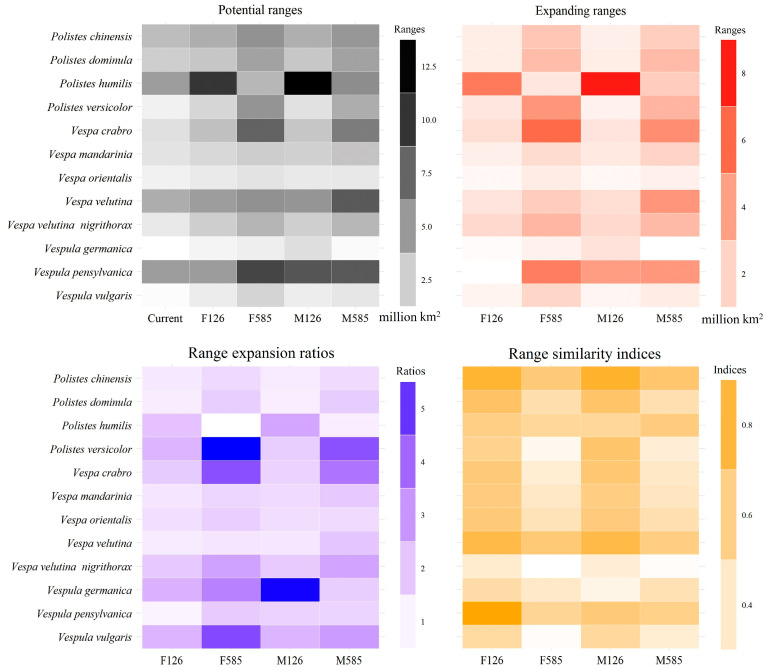
Potential ranges and range dynamics of the 12 major invasive wasp species under future scenarios. The potential ranges are shown in grayscale. The range dynamics are indicated by expanding ranges in redscale, the range expansion ratios in bluescale, and the range similarity indices in yellowscale. Under most scenarios, *Polistes dominula*, *Vespa crabro*, and *Vespula germanica* were projected to show larger potential ranges than the other species; *Vespa velutina nigrithorax* and *Polistes chinensis* were projected to have the higher range expansion ratios; *Polistes versicolor* and *P. chinensis* were projected to have lower range similarity indices; and *Vespa velutina* and *Polistes dominula* were projected to have larger expanding ranges.

The potential ranges of invasive wasps also varied with species identity (Supplementary Materials S9). For instance, the ranges of *Vespula vulgaris* under the F126 scenario were mainly projected in Europe, covering 4.05 million km^2^ (Figure 5, Supplementary Materials S9), while those of *Vespa mandarinia* were mainly identified in East China, the Korean Peninsula, and Japan, where they covered 1.30 million km^2^ (Figure 5, Supplementary Materials S9). Additionally, the potential ranges were also scenario-specific (Figure 5, Supplementary Materials S9). Under the scenario of F585, the range of *Vespa velutina nigrithorax* was mostly identified in the European continent, East China, and in the state of Michigan, USA, and covered 5.24 million km^2^, whereas those under the M126 scenario occurred principally in Europe, covering 1.69 million km^2^ (Figure 5, Supplementary Materials S9).

The range sizes of the 12 invasive wasp species spanned 0.32 to 4.83, 0.78 to 9.88, 1.16 to 12.91, 1.12 to 9.13, and 0.59 to 8.15 million km^2^ under current conditions and the F126, M126, F585, and M585 scenarios, respectively (Figure 5). The largest range was identified for *Vespula germanica* under the M126 scenario (12.91 million km^2^), and the smallest one was identified for *Polistes humilis* under the current scenario (0.32 million km^2^).

Our paired-samples t-test showed that the current ranges of the 12 invasive wasp species were smaller than those under future scenarios (paired-samples t-test: P equal to 0.02, 0.003, 0.02, and less than 0.001 under the F126, F585, M126, and M585 scenarios, respectively). Under all five scenarios, both *Polistes dominula* and *Vespa crabro* appeared five times in the list of the three largest ranges (Figure 5, Supplementary Materials S9), and both of them were mainly detected in Europe and the United States, while the corresponding frequencies for *Vespa mandarinia* and *Polistes chinensis* were five in the three smallest ranges, with the former being scattered across the six continents and the latter being mainly projected in East Asia (Figure 5, Supplementary Materials S9). 

**Figure 6:**
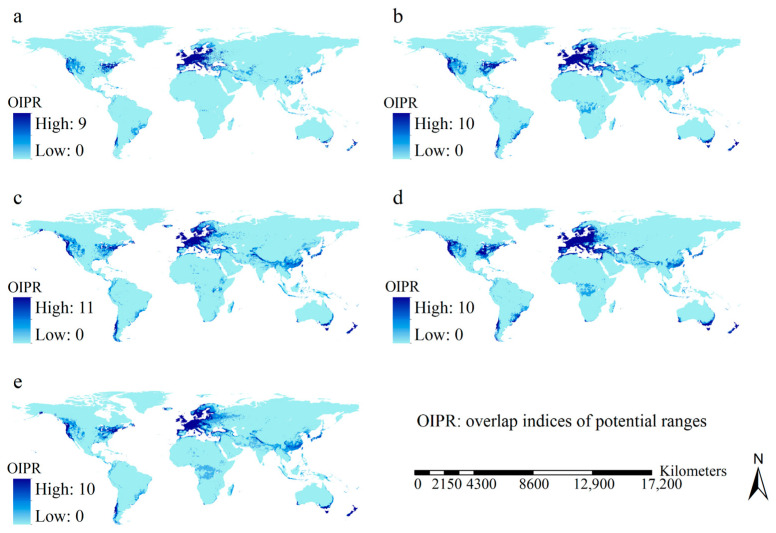
Overlap indices of potential ranges of the 12 major invasive wasp species: (**a**) overlap indices of potential ranges under the current scenario; (**b**) range overlap indices under F126; (**c**) range overlap indices under F585; (**d**) range overlap indices under M126; (**e**) range overlap indices under M585. High overlap indices of potential ranges were mainly projected in Europe, the eastern part of the USA, a region to the west of the Cascade Mountain Range in the USA, the eastern part of China, Japan, the southeast part of Australia, New Zealand, Argentina, and the southeastern part of Brazil.

Although the potential ranges of the 12 invasive wasp species were evidently species- and scenario-specific, their high overlaps under all five scenarios were mainly detected in Europe, eastern USA, a region to the west of the Cascade Mountain Range in the USA, East China, most of Japan, and southeastern Australia and Brazil, along with most of Japan, New Zealand, and Argentina (Figure 6).

### 3.5. Range Dynamics of the Invasive Wasps

Species-specific range dynamics were found for the 12 invasive wasp species (Figure 5). For instance, under the F126 scenario, the range expansion ratios (range similarity indices) for *Polistes chinensis* and *Vespula pensylvanica* were 2.36 (0.52) and 1.15 (0.70), respectively (Figure 5). Additionally, the wasps’ range dynamics also differed among scenarios. For example, under both the F585 and M126 scenarios, the range expansion ratios and range similarity indices for *Vespa velutina* were 4.46 (0.36) and 1.70 (0.66). *Vespa velutina nigrithorax* and *Polistes chinensis* had frequencies of three and four, respectively, in the lists of the three highest range expansion ratios under the four future scenarios (Figure 5). Under the four future scenarios, both *Vespa crabro* and *Vespula vulgaris* appeared thrice in the lists of the three lowest range expansion ratios and four times in the lists of the three highest range similarity indices (Figure 5). Both *Polistes versicolor* and *P. chinensis* had frequencies of four in the lists of the three lowest range similarity indices under the four future scenarios (Figure 5). 

All 12 major invasive wasp species were expected to expand their ranges under most future scenarios (i.e., all range expansion ratios were > 1, except those under the F585 scenario for *Vespula germanica*; Figure 5). The majority of the range similarity indices for the invasive wasp species under the F126 and M126 scenarios were above 0.5, i.e., 91.7% and 83.3% of the 12 species were projected to have range similarity indices > 0.5 under the scenarios, respectively (Figure 5). However, under the F585 as well as the M585 scenario, the majority of range similarity indices were under 0.5, i.e., 66.7% of the 12 species were projected to have range similarity indices < 0.5 under both scenarios. 

**Figure 7:**
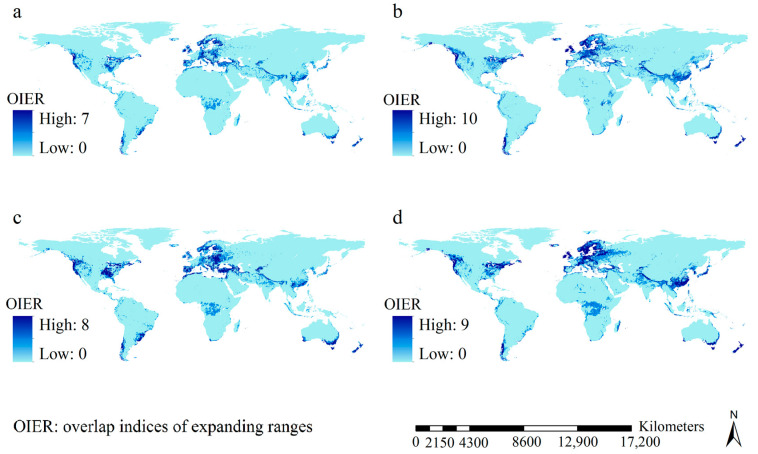
Overlap indices of expanding ranges of the 12 major invasive wasps. (**a**) overlap indices of expanding ranges of expanding ranges under F126 scenarios; (**b**) overlap indices of expanding ranges of expanding ranges under F585 scenarios; (**c**) overlap indices of expanding ranges under M126 scenarios; (**d**) overlap indices of expanding ranges under M585 scenarios. High values of overlapping indices of expanding ranges in Europe, northeastern and northwestern parts of the United States of America, southeastern and southwestern parts of Canada, Southeast China, New Zealand, southeastern coastline regions of Australia, Japan and southern part of Chile.

Notably, both *Vespa velutina* and *Polistes dominula* were expected to have frequencies of three in the lists of the top three expanding ranges under all future scenarios, while *P. humilis* and *Vespa mandarinia* had frequencies of three and four in the lists of the three smallest expanding ranges (Figure 5). Additionally, *Vespa velutina* was expected to have the largest expanded ranges under all future scenarios, which were mainly identified in Asia and Europe (Supplementary Materials S9). Although the spatial patterns for the overlap indices of expanding ranges did vary slight across the scenarios, we detected high values of overlapping indices of expanding ranges in Europe, northeastern and northwestern USA, southeastern and southwestern Canada, southeastern coastline regions of Australia, and southern Chile, along with Japan, Southeast China, and New Zealand (Figure 7). 

## 4. Discussion

By applying a unified approach, we developed 12 species distribution models to predict the global range shifts of invasive wasp species under current and future scenarios. We showed that all 12 species are projected to undergo substantial expansion in their potential ranges under most of the future scenarios. We also projected future increases in overlap indices of habitat suitability in more than 75% of the global land area. Therefore, our results suggest escalating threats of future invasion by these 12 invasive wasp species, leaving global apiculture at greater risk from their stronger impacts under future scenarios compared to current conditions.

Our study shows that climatic factors are projected to exhibit instrumental roles in shaping the range dynamics of the 12 invasive wasp species. This observation, to a certain extent, is supported by many studies [50,51,52,53]. Additionally, the sizes of their potential ranges under the future scenarios were larger than those under the current scenarios. Hence, mitigating future climate change could be among the essential approaches to combatting wasp invasions and lessening their adverse impacts on global apiculture. 

The relative influence of climatic and land-use predictors on species’ ranges may depend on spatial scales, i.e., stronger influences of climate factors occur on a large scale, while land-use factors are stronger on a small scale [54]. This argument is supported by our global-scale investigation of the range dynamics of the 12 studied invasive wasp species. However, work performed on a local scale in the Iberian Peninsula by Bessa et al. [25] and in the UK by Sumner et al. [16] showed that, relative to land-use factors, climatic factors exacted stronger and weaker effects on the range dynamics of invasive wasps, respectively. Therefore, uncertainty remains regarding the general applicability of Sirami et al.’s argument [26] to the range dynamics of the invasive wasps, and more case studies related to this topic are now needed.

It could be reasonably assumed that larger potential ranges, to a certain extent, imply higher spread risks for invasive species. Our studies showed that *Polistes dominula* and *Vespa crabro* were most frequently in the lists of the three largest ranges under all scenarios. Therefore, in terms of the sizes of potential ranges, these two species of invasive wasps pose the greatest invasion risk under current and future scenarios. They therefore deserve much more attention than the other wasp species. 

In this study, we created a range expansion ratio to measure the sizes of future ranges relative to those of current ones, and a higher range expansion ratio generally suggested a higher invasion potential in current and future scenarios. Our study demonstrated that *Polistes chinensis* and *Vespa velutina nigrithorax* had the highest frequencies in the lists of the three highest range expansion ratios under all future scenarios. That is, in the future, these two invasive wasp species could expand their ranges with greater magnitudes or ratios than the other invasive wasp species. Accordingly, it is imperative to pay more attention to them than the other wasp species when considering current and future scenarios. We also found that *Vespa velutina* and *Polistes dominula* underwent the greatest range expansions in most of the future scenarios, indicating that these two species are apt to substantially invade new regions. To control their future expansion, we should focus on their expanding ranges more than those of other invasive wasp species.

In our study, an index of range similarity was built to reflect the centroid dynamics in the potential ranges of invasive wasps. Lower range similarity indices indicate much stronger centroid shifts, suggesting that glaring shifts in priority regions should be incorporated into strategies tackling their invasions. Our results revealed that *Polistes versicolor* and *P. chinensis* had the lowest range expansion ratios under all future scenarios, which implied that their centroid shifts were the largest among the 12 invasive wasp species. So, to efficiently counter their future invasions, we should markedly shift our priority regions for action vis-à-vis their invasions. 

Of the 12 major invasive wasp species, only *Vespula vulgaris* is one of the 100 world’s worst invasive alien species (100WWIAS), suggesting its higher risk or invasiveness relative to other invasive species not listed among the 100WWIAS. Much attention is already being paid to understanding this wasp’s range dynamics [28,55,56,57]. Yet, surprisingly, in our study this invasive wasp was not the species with the largest potential ranges or expanding ranges, nor did it have the highest range expansion ratios. In other words, based on our results, this invasive wasp species does not pose a higher invasion risk than the other 11 wasp species studied. This contradiction might stem from the different measuring metrics used to infer invasion risk. We used range dynamics to assess invasion risk, while for the 100WWIAS list, other metrics were applied, e.g., impacts on global ecosystems, human health, and the apiculture industry [58,59,60].

Our study showed that, under all scenarios, high overlap indices for the potential ranges of the 12 invasive wasp species were mainly detected in Europe, eastern USA, and a region to the west of the Cascade Mountain Range in the USA. This pattern suggests that many more diverse invasive wasp species could find patches of suitable habitat in these regions than elsewhere. Because these regions could face higher threats from invasive wasps than other regions both now and in the future, they deserve much more attention than other regions. Our study also showed that high increases in the overlap indices of habitat suitability in the current and future scenarios were mainly projected in Northern Europe, Nepal, East China, and the eastern USA, implying that the magnitude of augmented invasion risk in these regions under the scenarios considered surpassed that in other regions. Therefore, stricter strategies against invasions of invasive wasps should be adopted in these four risk-prone regions in the future. We also found that the expanding ranges of the invasive wasps in the future included newly invaded regions. We detected high values of overlapping indices of expanding ranges mainly in Europe, northeastern and northwestern USA, southeastern and southwestern Canada, and so on. This result suggests that it would be prudent to tailor future strategies against prospective invasion by these wasp species by allocating more time and resources in these regions. 

Although wasp invasions can strongly impact ecosystems, biodiversity, and human health, their effects on apiculture have received disproportionately greater attention [3,4,6,60,61,62,63]. Our study forecasts that all 12 invasive wasp species will expand their ranges in the future, and the increase in their overlap indices of habitat suitability could affect more than 75% of the global land area in the future. Thus, beekeepers worldwide could face graver threats from invasive wasps in this century, implying that they should develop more efficient management and quarantine measures to counter the increasing impacts of invasive wasps in the future. Our study also showed that *Polistes dominula* and *Vespa crabro* attained the largest potential ranges under most of the scenarios, and their potential ranges were mainly detected in Europe and the United States. Hence, under current and future scenarios, these two invasive wasp species consistently pose great threats to beekeepers in these regions. Therefore, beekeepers should input much more time and energy to combat their impacts in these areas than those of other invasive wasp species. We also found that *Vespa velutina* had the largest expanding ranges under all future scenarios, which were mainly projected in some regions of Europe and Asia. This implies that beekeepers should adopt stricter quarantine measures against this wasp in the future than in current conditions because its threat in these regions does not yet exist. Further, our study showed that, under all the scenarios, high overlap indices for the potential ranges of the 12 major invasive wasp species were mainly detected in Europe and the United States, suggesting that beekeepers in these regions might face greater threats from the invasive wasps than in other regions and that they should devise much stricter management and prevention strategies against wasp invasions than elsewhere. Our study also projected substantial increases in the overlap indices of habitat suitability in the future, mainly in Northern Europe, Nepal, East China, and eastern USA, implying that beekeepers in these regions can expect considerably mounting impacts of wasps on apiculture. Therefore, they should increase their efforts to monitor and control wasp invasions in the future. 

## 5. Conclusions

In this study, we derived 12 species distribution models to project the range dynamics and invasion hotspots of 12 major invasive Vespidae wasp species via a unified approach, and we also assessed their impacts on global apiculture. We found that habitat suitability increased in most terrestrial parts of the world and that all underwent substantial range expansions, primarily induced by future climate changes. We also identified those invasive wasp species projected to have the largest potential ranges, highest range expansion ratios, and largest centroid shifts, along with the invasion hotspots of all major invasive wasp species, in the future. Altogether, the habitat suitability increases, range expansions, and centroid shifts of the invasive wasps suggest that global apiculture will be prone to worse impacts from them in the future.

## Data Availability

We have supplied all data in the Supplementary Materials.

## Supplementary Materials

The following supporting information can be downloaded at: https://www.mdpi.com/article/10.3390/insects15070546/s1, S1: The list of major invasive wasp species; S2: Importance of all variables in the full models; S3: Multicollinearity among all variables; S4: Algorithms used to build formal and final models; S5: Model performance assessment; S6: Variable importance in formal and final models; S7: Habitat suitability patterns of twelve invasive wasp species; S8: MSS thresholds used to determine ranges; S9: Ranges for the 12 major invasive wasp species.
