# Supplementary material for: Future Range Expansions of Invasive Wasps Suggest Their Increasing Impacts on Global Apiculture
Source: Insects. 2024 Jul 19;15(7):546. doi: 10.3390/insects15070546 (PMC11276961; doi:10.3390/insects15070546)
Supplement: Supplementary file 1 [file insects-15-00546-s001.zip › Supplemental material S1.pdf]

S1 The list of the major invasive wasp specie

Polistes dominula, *Polistes chinensis*, *Polistes versicolor*, *Polistes humilis*, *Vespula vulgaris*, *Vespula germanica*, *Vespula pensylvanica*, *Vespa velutina*, *Vespa mandarinia*, *Vespa velutina nigrithorax*, *Vespa orientalis* and *Vespa crabro.*

References

Addison, P., Veldtman, R., & Van Zyl, C. (2018). The invasive Vespidae in South Africa: potential management strategies and current status. African entomology, 26(2), 267-285.

Alaniz, A. J., Carvajal, M. A., & Vergara, P. M. (2021). Giants are coming? Predicting the potential spread and impacts of the giant Asian hornet (*Vespa mandarinia*, Hymenoptera: Vespidae) in the USA. Pest management science, 77(1), 104-112.

Beggs, J. (2001). The ecological consequences of social wasps (*Vespula spp.*) invading an ecosystem that has an abundant carbohydrate resource. Biological Conservation, 99(1), 17-28.

Beggs, J. R., Brockerhoff, E. G., Corley, J. C., Kenis, M., Masciocchi, M., Muller, F., ... & Villemant, C. (2011). Ecological effects and management of invasive alien Vespidae. BioControl, 56, 505-526.

Benadé, P. C., Veldtman, R., Samways, M. J., & Roets, F. (2014). Rapid range expansion of the invasive wasp *Polistes dominula* (Hymenoptera: Vespidae: Polistinae) and first record of parasitoids on this species and the native *Polistes marginalis* in the Western Cape Province of South Africa. African Entomology, 22(1), 220-225.

Bessa, A. S., Carvalho, J., Gomes, A., & Santarém, F. (2016). Climate and land-use drivers of invasion: predicting the expansion of *Vespa velutina nigrithorax* into the Iberian Peninsula. Insect Conservation and Diversity, 1(9), 27-37.

Bulgarella, M., Mieles, A. E., Rodríguez, J., Campaña, Y., Richardson, G. M., Keyzers, R. A., ... & Lester, P. J. (2022). Integrating biochemical and behavioral approaches to develop a bait to manage the invasive yellow paper wasp *Polistes versicolor* (Hymenoptera, Vespidae) in the Galápagos Islands. Neotropical Biodiversity, 8(1), 271-280.

Bulgarella, M., Baty, J. W., McGruddy, R., & Lester, P. J. (2023). Gene silencing for invasive paper wasp management: Synthesized dsRNA can modify gene expression but did not affect

Carisio, L., Cerri, J., Lioy, S., Bianchi, E., Bertolino, S., & Porporato, M. (2022). Impacts of the invasive hornet Vespa velutina on native wasp species: a first effort to understand population-level effects in an invaded area of Europe. Journal of Insect Conservation, 26(4), 663-671.

Cunningham‐Eurich, I., Kontou, D., Yordanova, M., Maeda‐Obregon, A., Favreau, E., Wang, J., ... & Sumner, S. (2023). Using citizen science data to assess the population genetic structure of the common yellowjacket wasp, Vespula vulgaris. Insect Molecular Biology, 32(6), 634-647.

Downing, H. (2012). Nest parameters of Polistes and Mischocyttarus species (Hymenoptera: Vespidae) before and after detection of the invasive wasp, *Polistes dominula* in western South Dakota and Wyoming. Journal of the Kansas Entomological Society, 85(1), 23-31.

Duthie, C., & Lester, P. J. (2013). Reduced densities of the invasive wasp, *Vespula vulgaris* (Hymenoptera: Vespidae), did not alter the invertebrate community composition of Nothofagus forests in New Zealand. Environmental entomology, 42(2), 223-230.

Gamboa, G. J., Greig, E. I., & Thom, M. C. (2002). The comparative biology of two sympatric paper wasps, the native Polistes fuscatus and the invasive *Polistes dominulus* (Hymenoptera, Vespidae). Insectes Sociaux, 49, 45-49.

Gorosito, C. A., & Cueto, V. R. (2024). Neotropical songbird chick predation by an invasive wasp, the German yellowjacket (*Vespula germanica*). Austral Ecology, 49(2), e13488.

Harrop, T. W., Guhlin, J., McLaughlin, G. M., Permina, E., Stockwell, P., Gilligan, J., ... & Dearden, P. K. (2020). High-quality assemblies for three invasive social wasps from the *Vespula* genus. G3: Genes, Genomes, Genetics, 10(10), 3479-3488.

Howse, M. W. F., Reason, A., Haywood, J., & Lester, P. J. (2024). Improving wasp control by identifying likely causes of eradication failure. Journal of Pest Science, 1-15.

Kishi, S., & Goka, K. (2017). Review of the invasive yellow-legged hornet, *Vespa velutina nigrithorax* (Hymenoptera: Vespidae), in Japan and its possible chemical control. Applied entomology and zoology, 52, 361-368.

Lester, P. J., & Beggs, J. R. (2019). Invasion success and management strategies for social *Vespula* wasps. Annual review of entomology, 64, 51-71.

Lester, P. J., O'Sullivan, D., & Perry, G. L. (2023). Gene drives for invasive wasp control: Extinction is unlikely, with suppression dependent on dispersal and growth rates. Ecological Applications, 33(7), e2912.

Lioy, S., Carisio, L., Manino, A., & Porporato, M. (2023). Climatic niche differentiation between the invasive hornet *Vespa velutina nigrithorax* and two native hornets in Europe, *Vespa crabro* and *Vespa orientalis*. Diversity, 15(4), 495.

Masciocchi, M., & Corley, J. (2013). Distribution, dispersal and spread of the invasive social wasp (*Vespula germanica*) in Argentina. Austral Ecology, 38(2), 162-168.

McGruddy, R., Howse, M. W., Haywood, J., Toft, R. J., & Lester, P. J. (2021). Nesting ecology and colony survival of two invasive *Polistes* wasps (Hymenoptera: Vespidae) in New Zealand. Environmental Entomology, 50(6), 1466-1473.

Melo, R., Masciocchi, M., & Corley, J. C. (2023). Allee effects in an invasive social wasp: an experimental study in colonies of Vespula germanica. Scientific Reports, 13(1), 16323.

Monceau, K., Bonnard, O. & Thiéry, D. *Vespa velutina*: a new invasive predator of honeybees in Europe. J Pest Sci 87, 1–16 (2014). https://doi.org/10.1007/s10340-013-0537-3

Moreyra, S., Lozada, M., Fernández-Arhex, V., & Pietrantuono, A. L. (2023). Polistes dominula (Hymenoptera: Vespidae) paper wasps detect native grasshoppers using olfactory cues. Austral Ecology, 48(8), 2041-2052.

Newsome, T., Cairncross, R., Cunningham, C. X., Spencer, E. E., Barton, P. S., Ripple, W. J., & Wirsing, A. J. (2024). Scavenging with invasive species. Biological Reviews, 99(2), 562-581.

Pereira, A. J., Pirk, G. I., & Corley, J. C. (2016). Foraging behavior interactions between two non-native social wasps, *Vespula germanica* and *V. vulgaris* (Hymenoptera: Vespidae): implications for invasion success?. Journal of Insect Science, 16(1), 78.

Rankin, E. E. W. (2021). Emerging patterns in social wasp invasions. Current opinion in insect science, 46, 72-77.

Reason, A., Felden, A., Bulgarella, M., & Lester, P. J. (2024). Population dynamics and prey community of the invasive paper wasp *Polistes chinensis* (Hymenoptera: Vespidae) in a protected coastal habitat in New Zealand. Austral Entomology.

Rojas-Nossa, S. V., O'Shea-Wheller, T. A., Poidatz, J., Mato, S., Osborne, J., & Garrido, J. (2023). Predator and Pollinator? An invasive hornet alters the pollination dynamics of a native plant. Basic and Applied Ecology, 71, 119-128.

Rothman, J. A., Loope, K. J., McFrederick, Q. S., & Wilson Rankin, E. E. (2021). Microbiome of the wasp Vespula pensylvanica in native and invasive populations, and associations with Moku virus. Plos one, 16(7), e0255463.

Sankovitz, M., Loope, K. J., Wilson Rankin, E. E., & Purcell, J. (2023). Unequal Reproduction Early in a Social Transition: Insights from Invasive Wasps. The American Naturalist, 201(2), 241-255.

Schmack, J. M., Lear, G., Astudillo-Garcia, C., Boyer, S., Ward, D. F., & Beggs, J. R. (2021). DNA metabarcoding of prey reveals spatial, temporal and diet partitioning of an island ecosystem by four invasive wasps. Journal of Applied Ecology, 58(6), 1199-1211.

Schmack, J. M., Schleuning, M., Ward, D. F., & Beggs, J. R. (2020). Biogeography and anthropogenic impact shape the success of invasive wasps on New Zealand's offshore islands. Diversity and Distributions, 26(4), 441-452.

Spencer, E. E., Barton, P. S., Ripple, W. J., & Newsome, T. M. (2020). Invasive European wasps alter scavenging dynamics around carrion. Food Webs, 24, e00144.

Stratford, J. E., Stratford, F. M., Brown, R. L., & Oi, C. A. (2024). Nest visitors of *Vespula* wasps and their potential use for biological control in an invaded range. Journal of Pest Science, 97(1), 445-453.

Veldtman, R., Addison, P., & Tribe, G. D. (2012). Current status and potential future impact of invasive vespid wasps (*Vespula germanica* and *Polistes dominulus*) in South Africa.

Villemant C, Barbet-Massin M, Perrard A, Muller F, Gargominy O, Jiguet F, Rome Q. Predicting the invasion risk by the alien bee-hawking Yellow-legged hornet Vespa velutina nigrithorax across Europe and other continents with niche models. Biol. Conserv. 2011: 144(9): 2142-2150.
