# Supplementary material for: Future Range Expansions of Invasive Wasps Suggest Their Increasing Impacts on Global Apiculture
Source: Insects. 2024 Jul 19;15(7):546. doi: 10.3390/insects15070546 (PMC11276961; doi:10.3390/insects15070546)
Supplement: Supplementary file 1 [file insects-15-00546-s001.zip › Supplemental Material S7.pdf]

S7 Habitat suitability maps of the invasive wasps

Polistes dominula

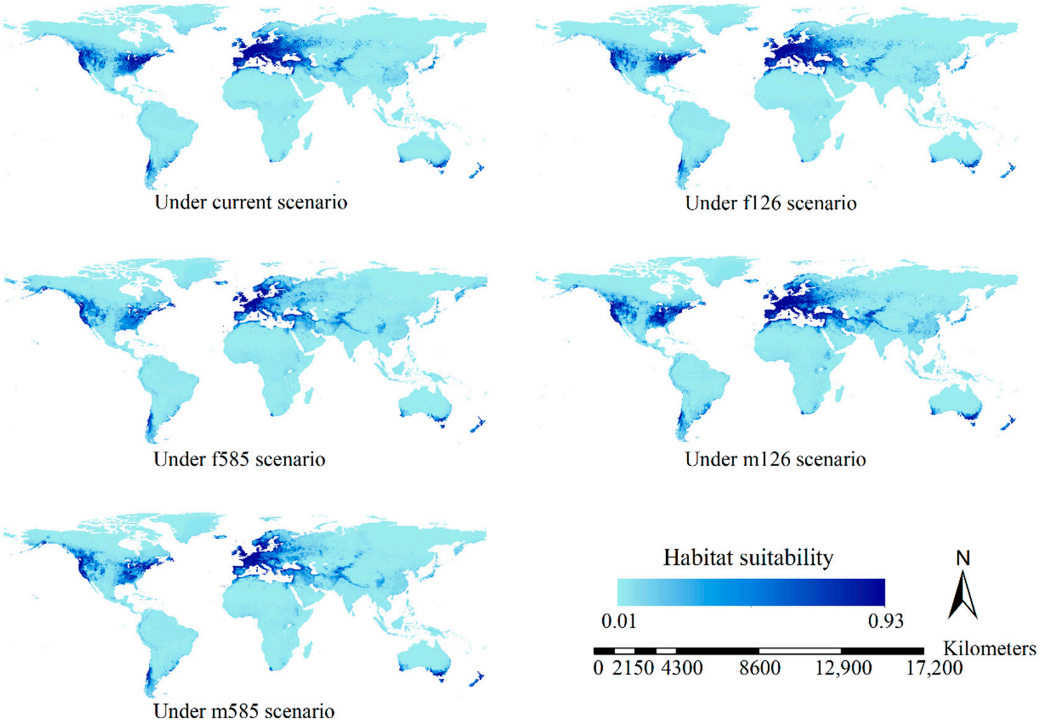

Polistes chinensis

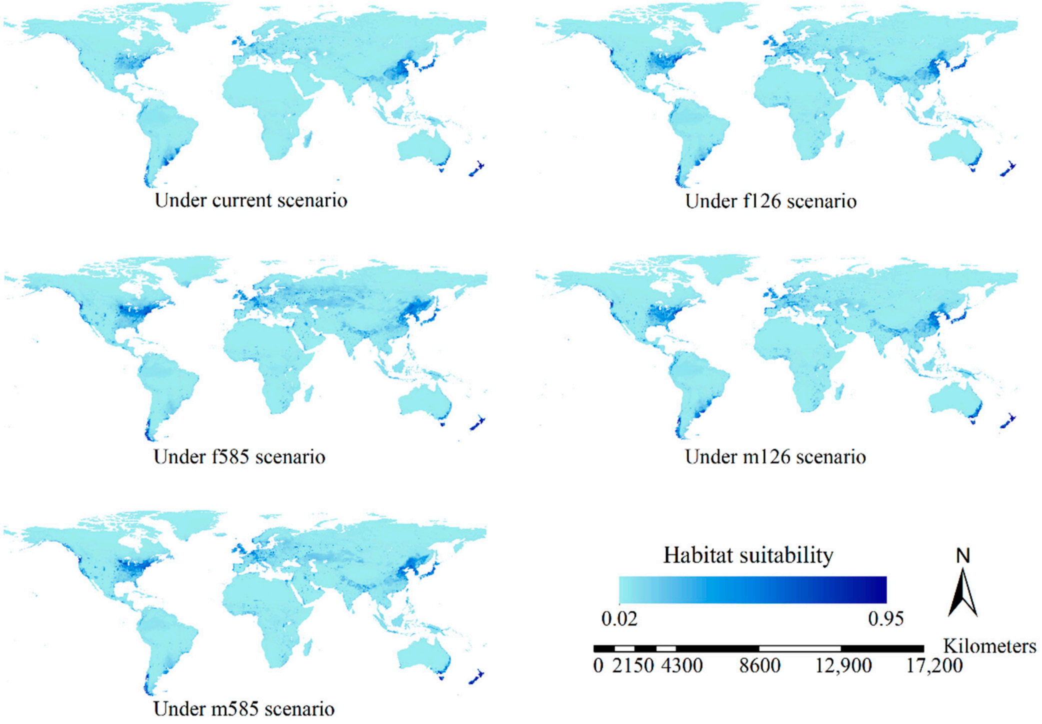

Polistes versicolor

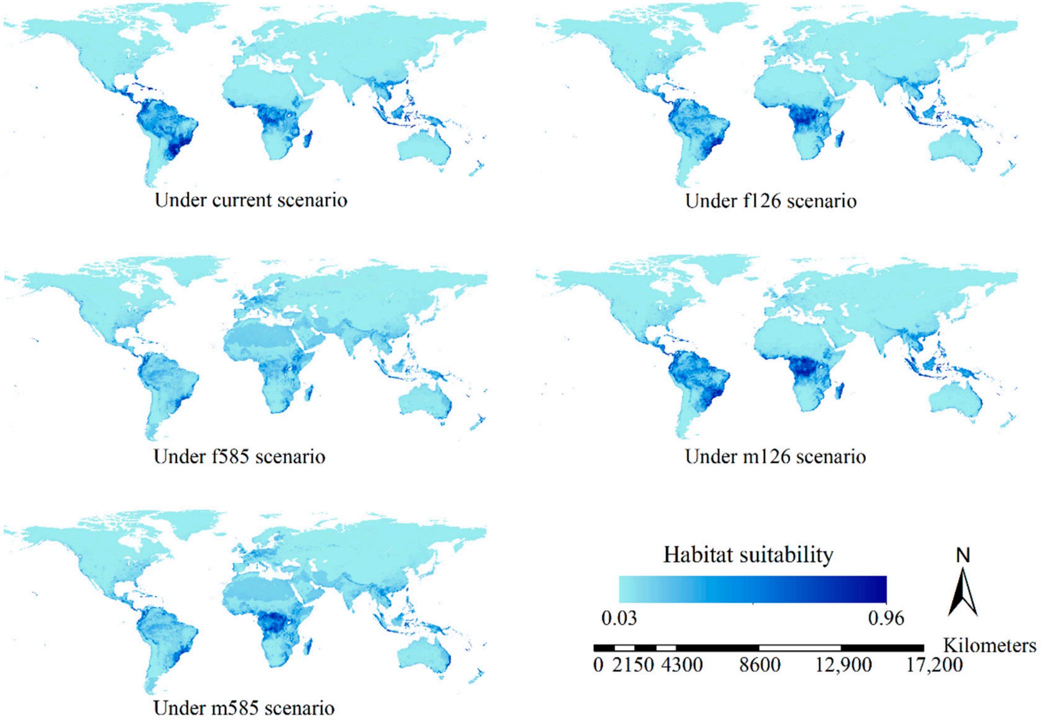

Polistes humilis

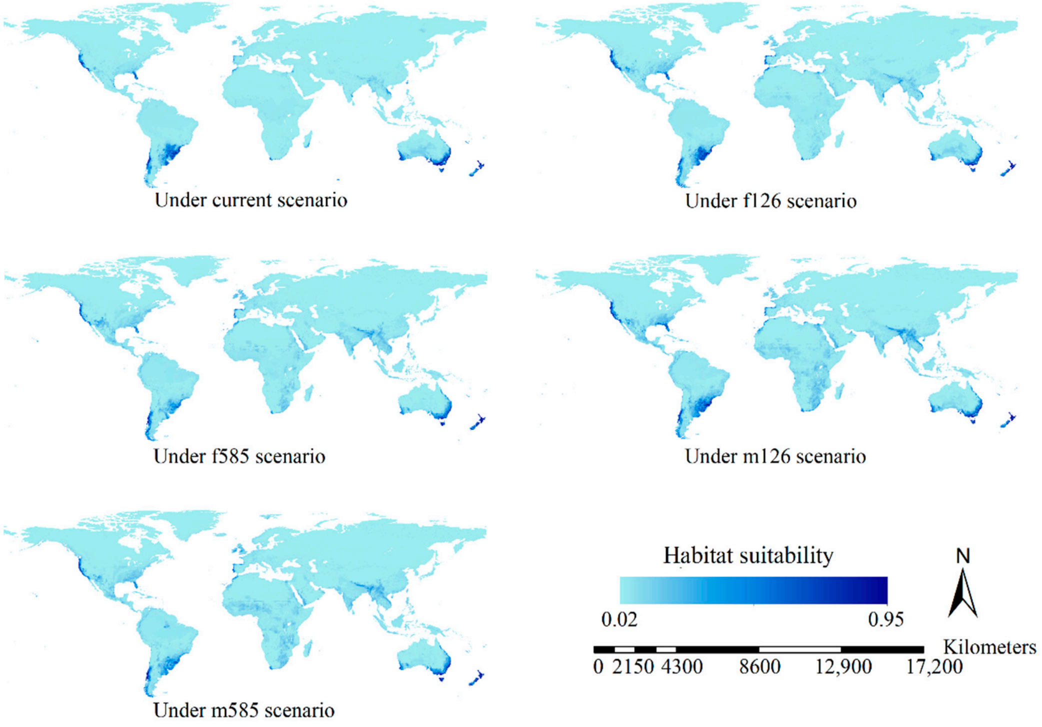

Vespula vulgaris

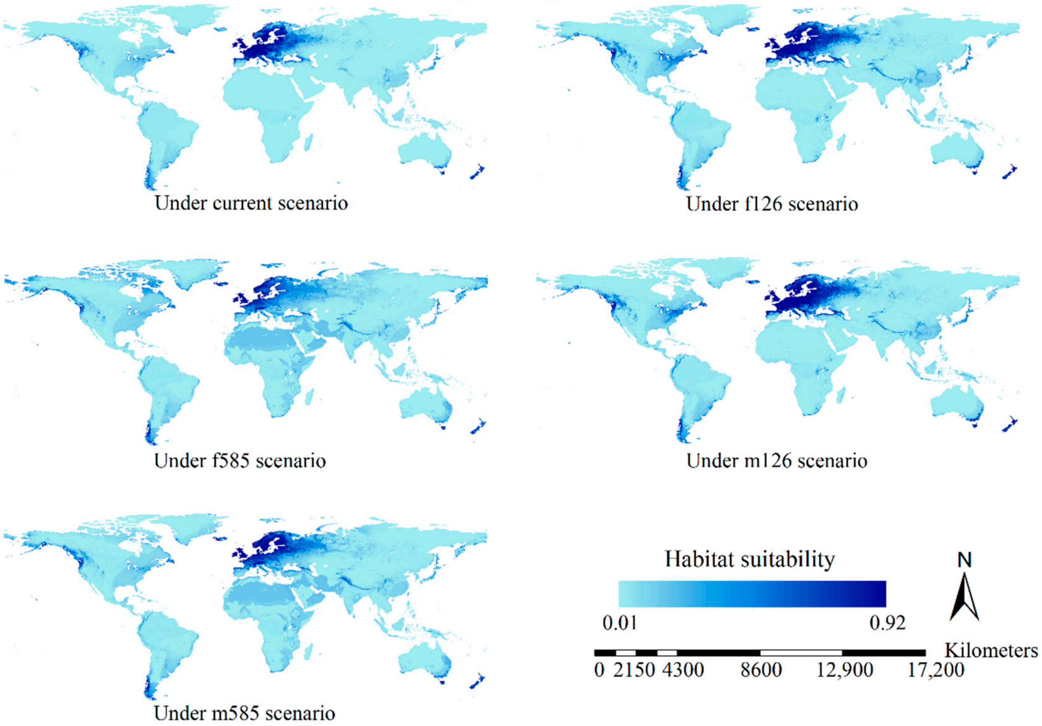

Vespula germanica

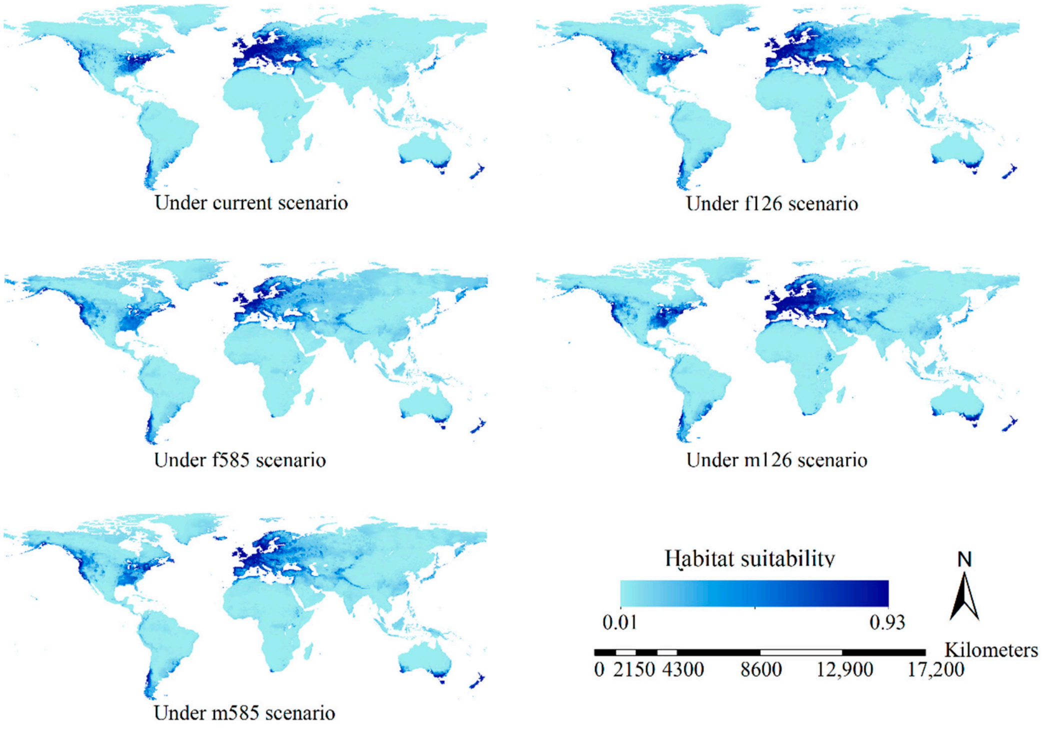

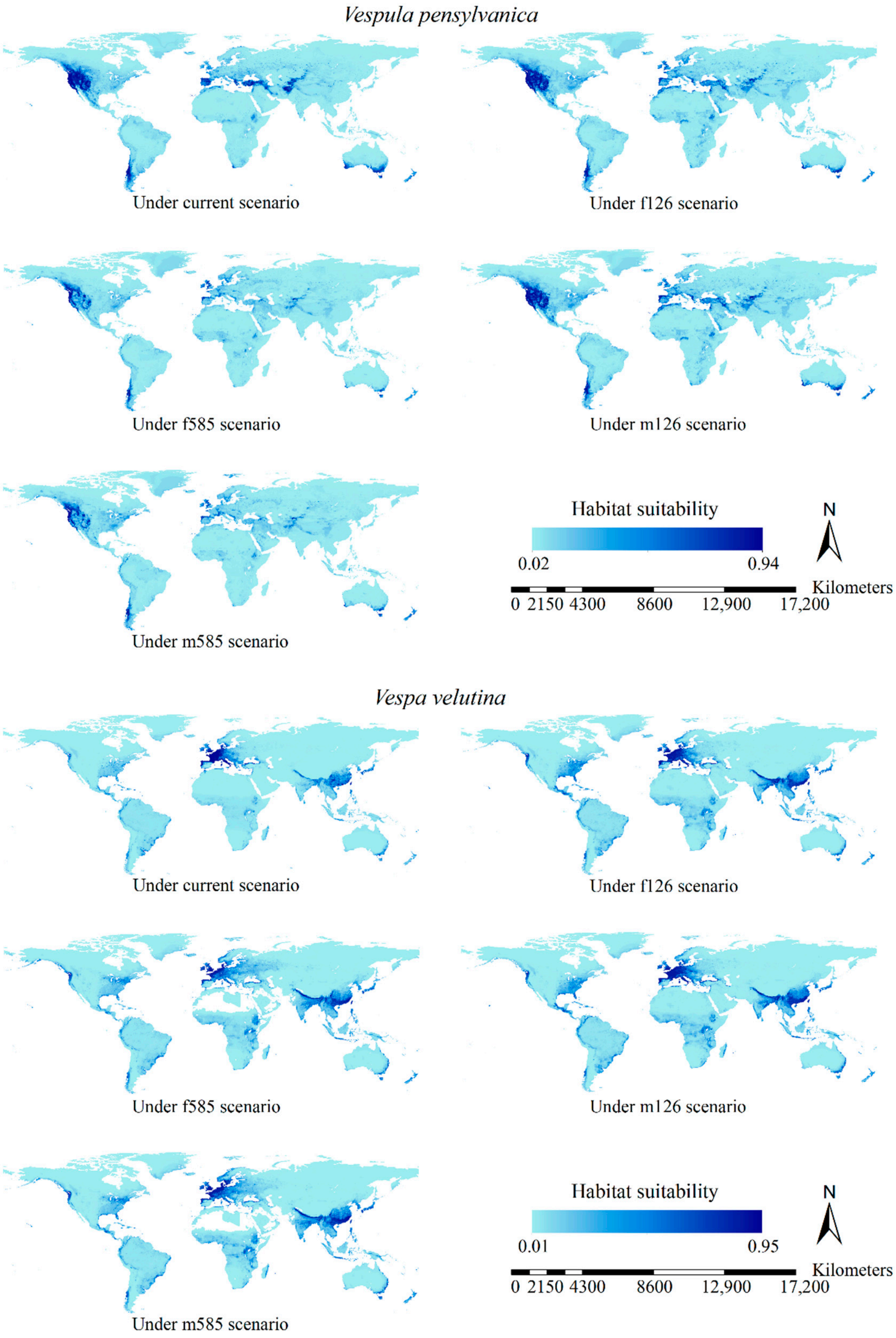

Vespula pensylvanica
Under current scenario
Under f126 scenario
Under f585 scenario
Under m126 scenario
Under m585 scenario
Habitat suitability
0.02
0.94
N
Kilometers
0 2150 4300 8600 12,900 17,200
Vespa velutina
Under current scenario
Under f126 scenario
Under f585 scenario
Under m126 scenario
Under m585 scenario
Habitat suitability
0.01
0.95
N
Kilometers
0 2150 4300 8600 12,900 17,200

Vespa mandarinia

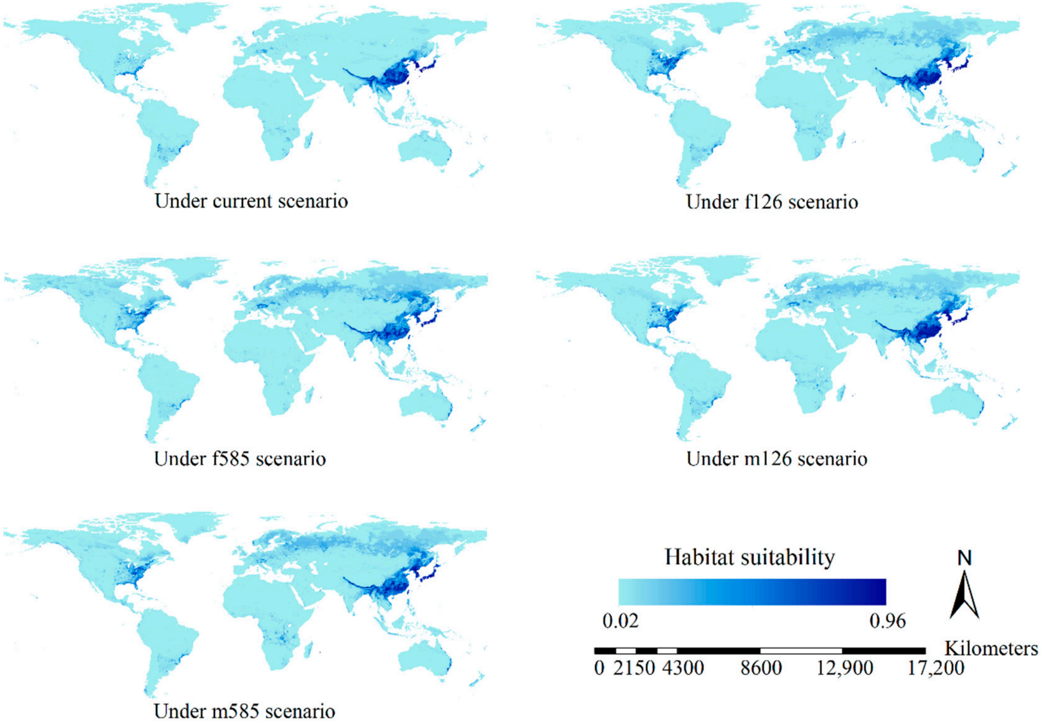

Vespa velutina nigrithorax

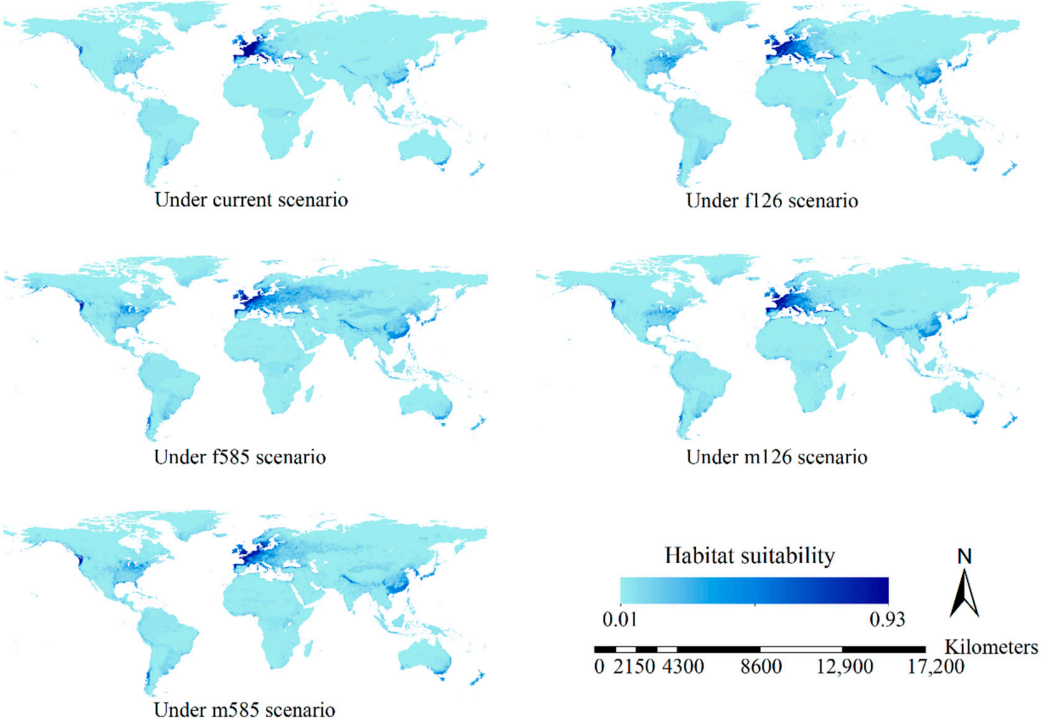

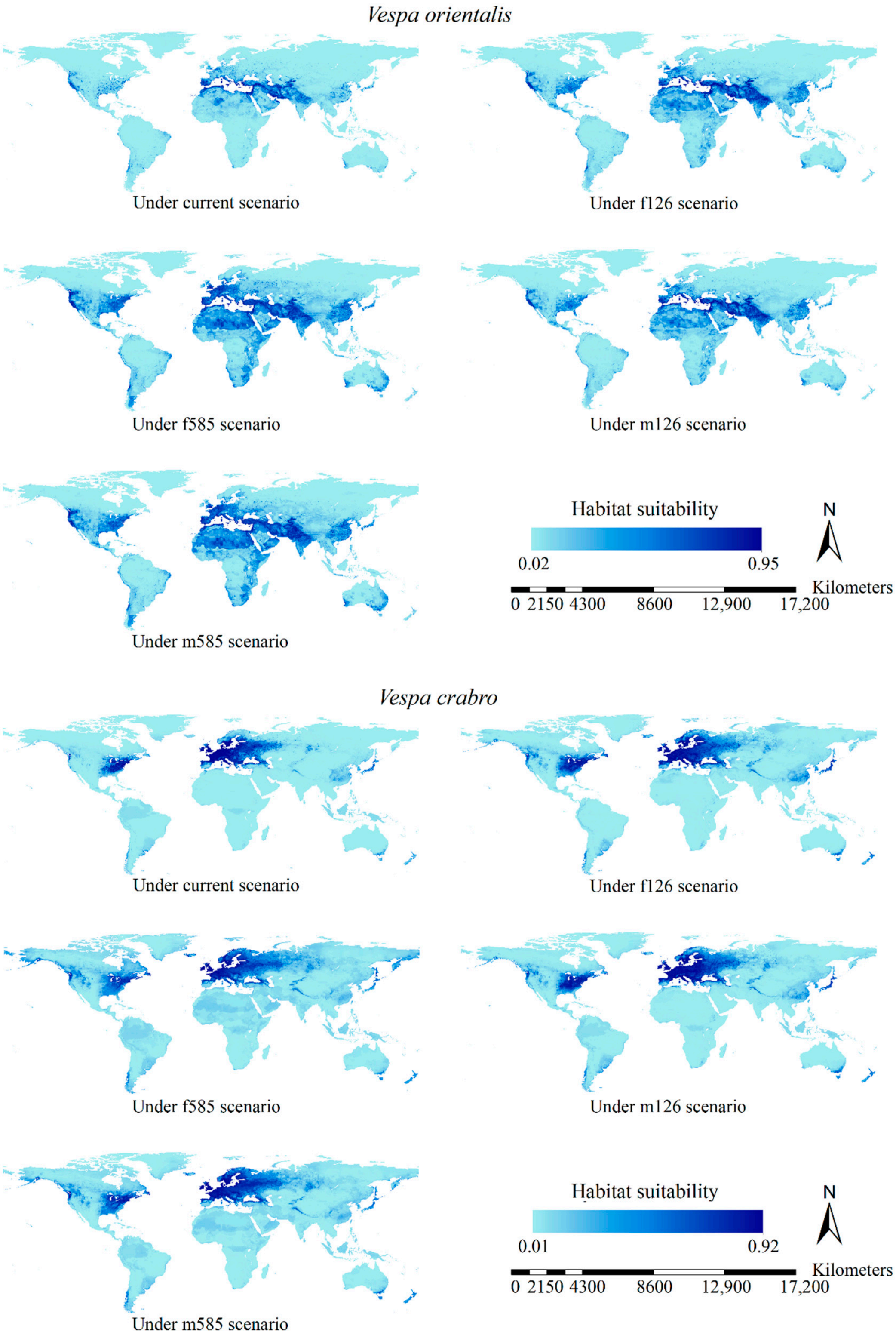

Vespa orientalis
Under current scenario
Under f126 scenario
Under f585 scenario
Under m126 scenario
Under m585 scenario
Habitat suitability
0.02
0.95
N
Kilometers
0 2150 4300 8600 12,900 17,200
Vespa crabro
Under current scenario
Under f126 scenario
Under f585 scenario
Under m126 scenario
Under m585 scenario
Habitat suitability
0.01
0.92
N
Kilometers
0 2150 4300 8600 12,900 17,200
