# Supplementary material for: Future Range Expansions of Invasive Wasps Suggest Their Increasing Impacts on Global Apiculture
Source: Insects. 2024 Jul 19;15(7):546. doi: 10.3390/insects15070546 (PMC11276961; doi:10.3390/insects15070546)
Supplement: Supplementary file 1 [file insects-15-00546-s001.zip › Supplemental Material S9.pdf]

S9 Ranges of the major 12 invasive wasps

Polistes dominula

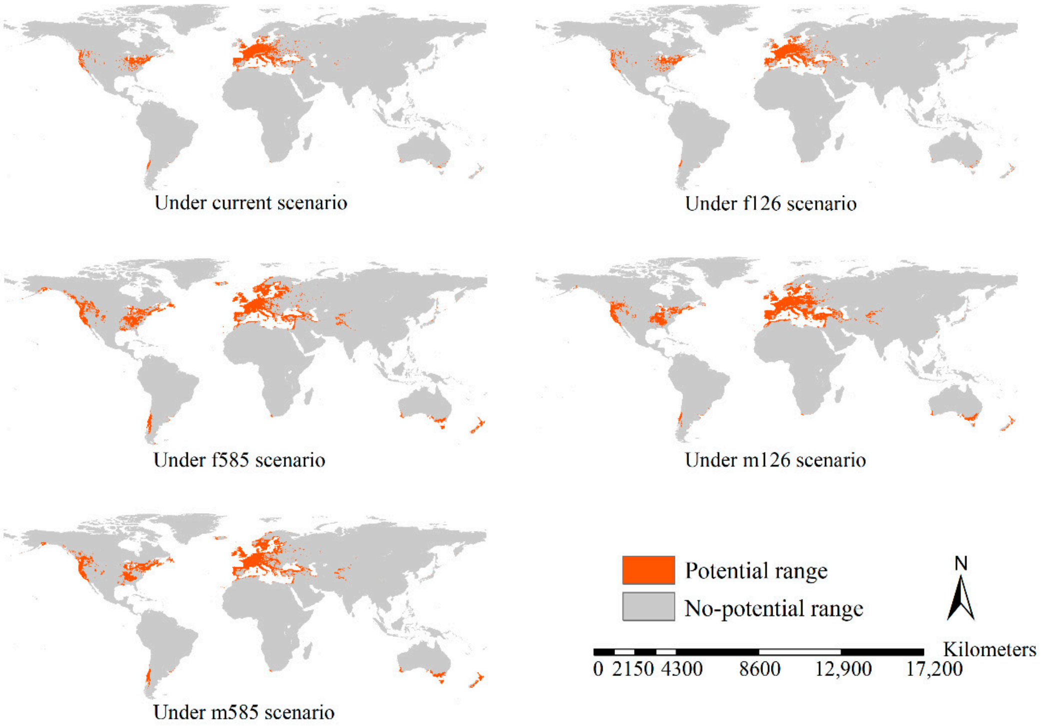

Polistes chinensis

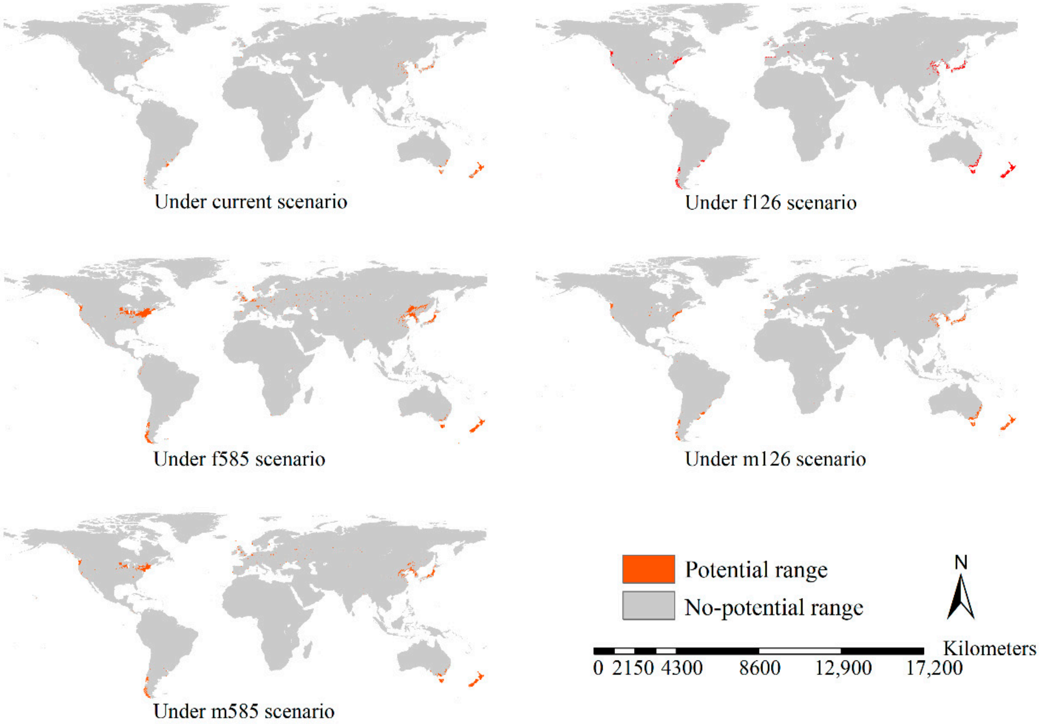

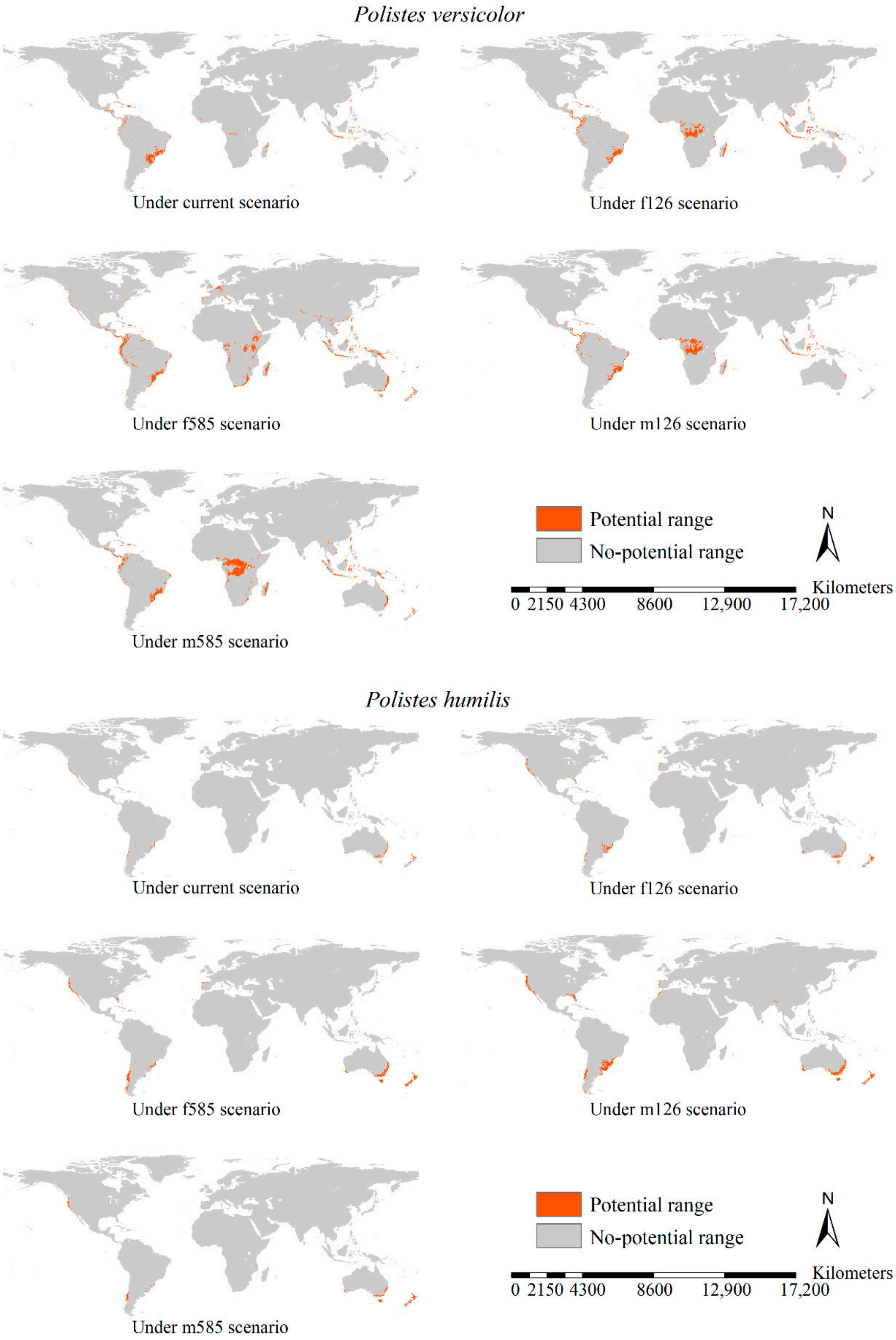
Polistes versicolor
Under current scenario
Under f126 scenario
Under f585 scenario
Under m126 scenario
Under m585 scenario
Potential range
No-potential range
N
Kilometers
0 2150 4300 8600 12,900 17,200
Polistes humilis
Under current scenario
Under f126 scenario
Under f585 scenario
Under m126 scenario
Under m585 scenario
Potential range
No-potential range
N
Kilometers
0 2150 4300 8600 12,900 17,200

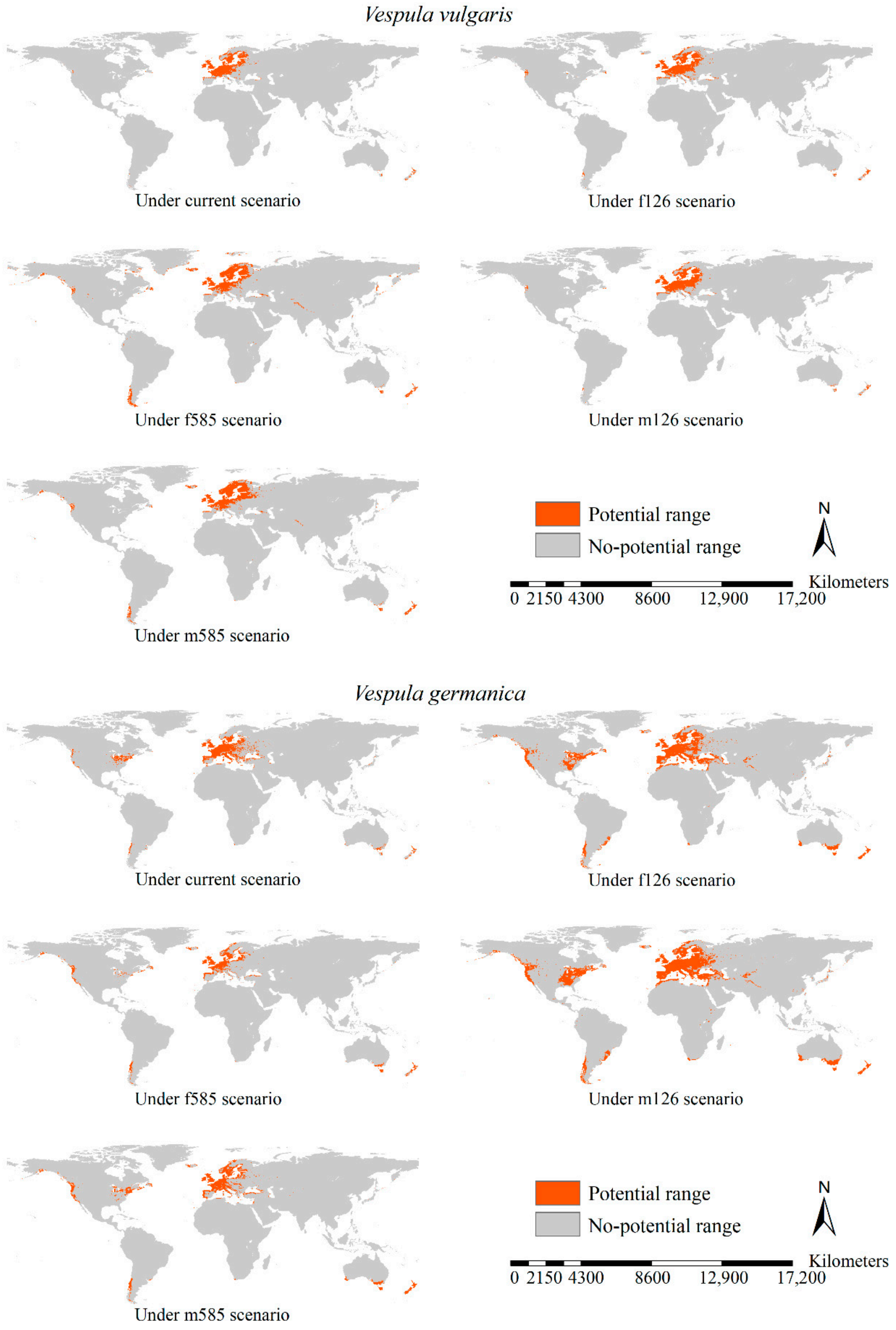
Vespula vulgaris
Under current scenario
Under f126 scenario
Under f585 scenario
Under m126 scenario
Under m585 scenario
Potential range
No-potential range
N
Kilometers
0 2150 4300 8600 12,900 17,200
Vespula germanica
Under current scenario
Under f126 scenario
Under f585 scenario
Under m126 scenario
Under m585 scenario
Potential range
No-potential range
N
Kilometers
0 2150 4300 8600 12,900 17,200

Vespula pensylvanica

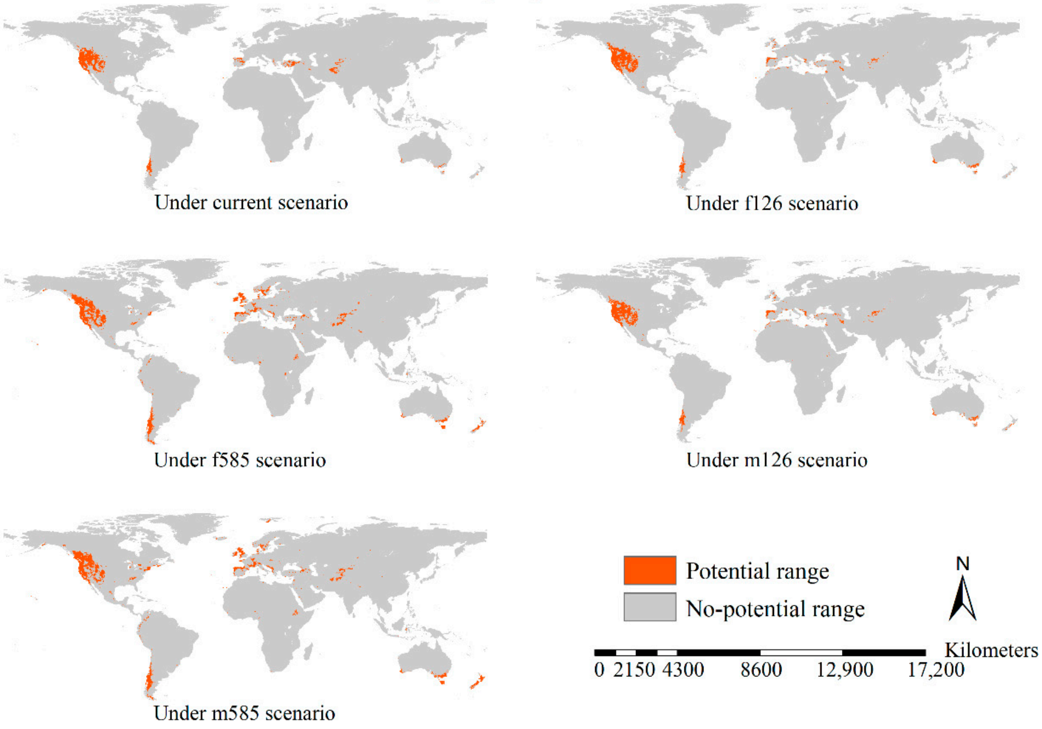

Vespa velutina

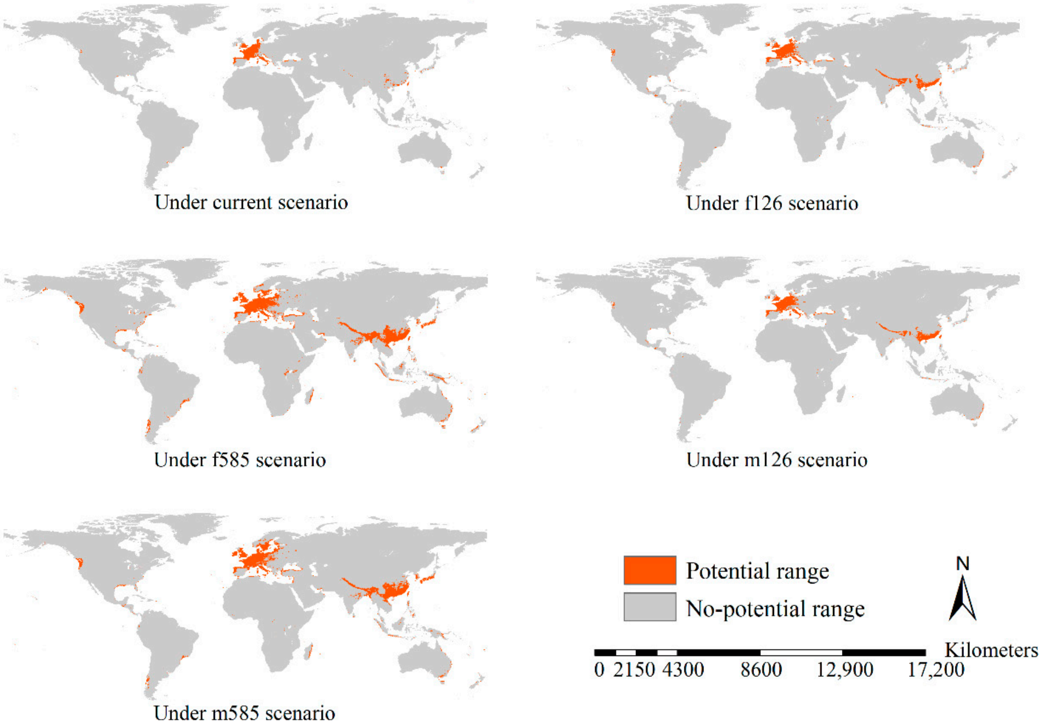

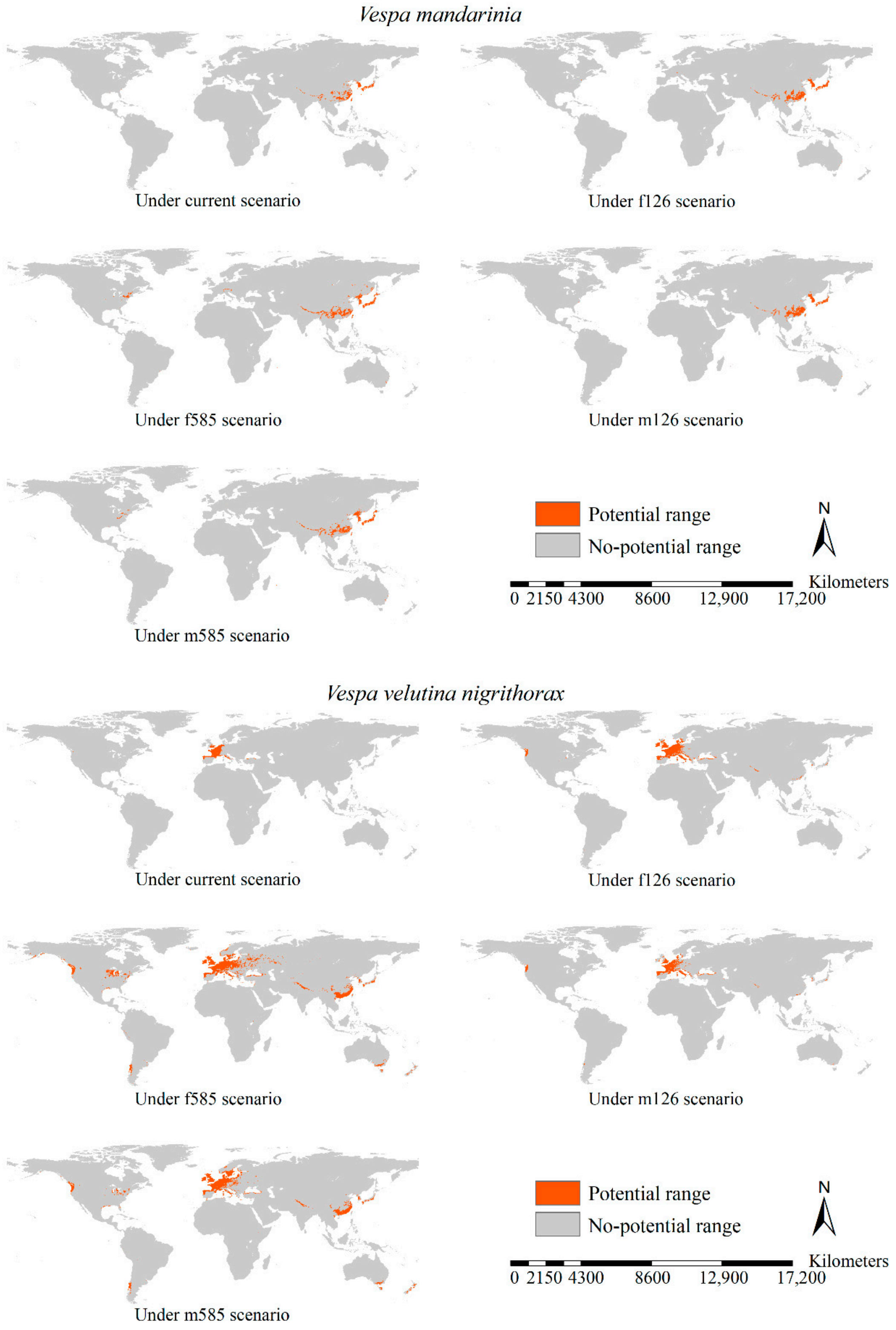
Vespa mandarinia
Under current scenario
Under f126 scenario
Under f585 scenario
Under m126 scenario
Under m585 scenario
Potential range
No-potential range
N
Kilometers
0 2150 4300 8600 12,900 17,200
Vespa velutina nigrithorax
Under current scenario
Under f126 scenario
Under f585 scenario
Under m126 scenario
Under m585 scenario
Potential range
No-potential range
N
Kilometers
0 2150 4300 8600 12,900 17,200

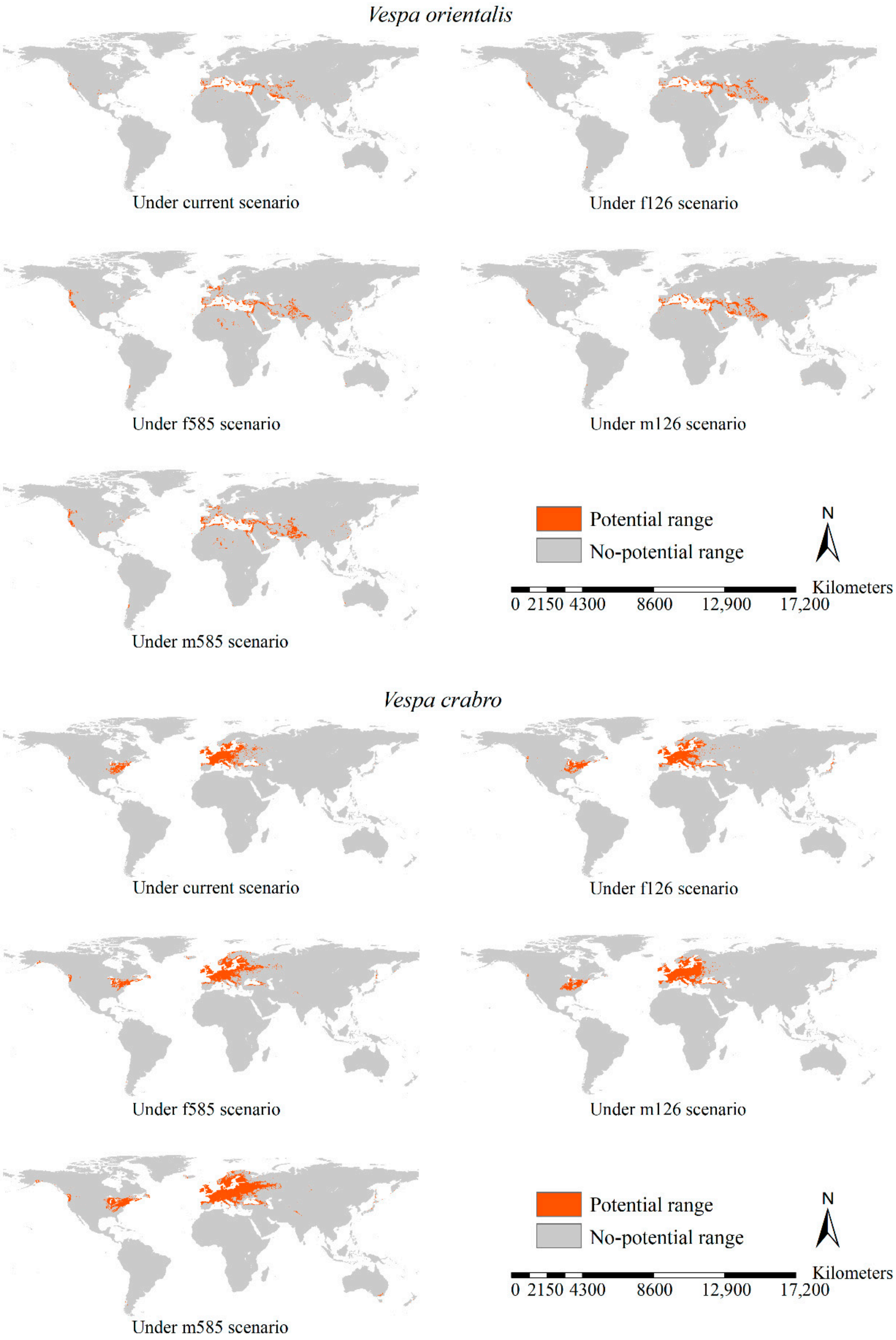

Vespa orientalis
Under current scenario
Under f126 scenario
Under f585 scenario
Under m126 scenario
Under m585 scenario
Potential range
No-potential range
N
Kilometers
0 2150 4300 8600 12,900 17,200
Vespa crabro
Under current scenario
Under f126 scenario
Under f585 scenario
Under m126 scenario
Under m585 scenario
Potential range
No-potential range
N
Kilometers
0 2150 4300 8600 12,900 17,200
